# Revealing stable SNPs and genomic prediction insights across environments enhance breeding strategies of productivity, defense, and climate-adaptability traits in white spruce

**DOI:** 10.1038/s41437-025-00747-z

**Published:** 2025-02-12

**Authors:** Eduardo P. Cappa, Charles Chen, Jennifer G. Klutsch, Jaime Sebastian-Azcona, Blaise Ratcliffe, Xiaojing Wei, Letitia Da Ros, Yang Liu, Sudarshana Reddy Bhumireddy, Andy Benowicz, Shawn D. Mansfield, Nadir Erbilgin, Barb R. Thomas, Yousry A. El-Kassaby

**Affiliations:** 1 Instituto Nacional de Tecnología Agropecuaria (INTA), Instituto de Recursos Biológicos, Centro de Investigación en Recursos Naturales, De Los Reseros y Dr. Nicolás Repetto s/n, 1686 Hurlingham Buenos Aires, Argentina; 2 Consejo Nacional de Investigaciones Científicas y Técnicas (CONICET), Buenos Aires, Argentina; 3 Department of Biochemistry and Molecular Biology, Oklahoma State University, Stillwater, OK 74078 USA; 4 Department of Renewable Resources, University of Alberta, 442 Earth Sciences Bldg., Edmonton, AB T6G 2E3 Canada; 5 Department of Forest and Conservation Sciences, Faculty of Forestry, University of British Columbia, Vancouver, BC V6T 1Z4 Canada; 6 Department of Wood Science, Faculty of Forestry, University of British Columbia, Vancouver, BC V6T 1Z4 Canada; 7 Department of Biological Sciences, University of Alberta, P217 Biological Sciences Building, Edmonton, AB T6G 2E9 Canada; 8 Forest Stewardship and Trade Branch, Alberta Forestry and Parks, Edmonton, AB T6H 5T6 Canada; 9 Department of Botany, Faculty of Science, University of British Columbia, Vancouver, BC V6T 1Z4 Canada; 10 Present Address: Natural Resources Canada, Canadian Forest Service, Northern Forestry Centre, Edmonton, AB T6H 3S5 Canada; 11 Present Address: Irrigation and Crop Ecophysiology Group, Instituto de Recursos Naturales y Agrobiología de Sevilla, Avenida Reina Mercedes, 10, 41012 Sevilla, Spain; 12 Present Address: Department of Chemistry, University of Saskatchewan, Saskatoon, SK S7N 5E2 Canada

**Keywords:** Plant breeding, Genomics

## Abstract

Exploring the relationship between phenotype, genotype, and environment is essential in quantitative genetics. Considering the complex genetic architecture of economically important traits, integrating genotype-by-environment interactions in a genome-wide association (GWAS) and genomic prediction (GP) framework is imperative. This integration is crucial for identifying robust markers with stability across diverse environments and improving the predictive accuracy of individuals’ performance within specific target environments. We conducted a multi-environment GWAS and GP analysis for 30 productivity, defense, and climate-adaptability traits on 1540 white spruce trees from Alberta, Canada, genotyped for 467,224 SNPs and growing across three environments. We identified 563 significant associations (*p*-value < 1.07 ×10^−05^) across the studied traits and environments, with 105 SNPs showing overlapping associations in two or three environments. Wood density, myrcene, total monoterpenes, α-pinene, and catechin exhibited the highest overlap (>50%) across environments. Gas exchange traits, including intercellular CO_2_ concentration and intrinsic water use efficiency, showed the highest number of significant associations (>38%) but less stability (<1.2%) across environments. Predictive ability (PA) varied significantly (0.03–0.41) across environments for 20 traits, with stable carbon isotope ratio having the highest average PA (0.36) and gas exchange traits the lowest (0.07). Only two traits showed differences in prediction bias (PB) across environments, with 80% of site-trait PB falling within a narrow range (0.90 to 1.10). Integrating multi-environment GWAS and GP analyses proved useful in identifying site-specific markers, understanding environmental impacts on PA and PB, and ultimately providing indirect insights into the environmental factors that influenced this white spruce breeding program.

## Introduction

White spruce (*Picea glauca* (Moench) Voss), a widely distributed tree species across Canada, northeastern USA and Alaska, is considered a keystone species in boreal forest ecosystems and a valuable resource for the commercial timber industry (Rweyongeza et al. 2007; Rweyongeza 2011). White spruce is also known for its remarkable adaptability, growing in diverse environmental conditions, varying soil types, cold climates, as well as moist and dry environments (OECD 2006). Given its wide ecological adaptability and economic importance, understanding the quantitative genetic underpinnings of trait variability related to productivity, growth, and resilience to biotic and abiotic stresses across environments, especially in the context of rapidly changing climate, is crucial for establishing sustainable forest management practices.

Genome-wide association studies (GWAS) and genomic prediction (GP) have emerged as powerful tools for dissecting complex traits and enhancing breeding efforts in several animal and plant species, including forest trees (Grattapaglia et al. 2018; Lebedev et al. 2020; Ahmar et al. 2021; Grattapaglia 2022; Isik 2022). Through whole-genome exploration, GWAS enables unraveling of a trait’s architecture, including the number of genetic variants influencing a particular quantitative trait, and understanding alleles’ differential effects on phenotypes and the linkage with the causal genes (Gianola 2013). On the other hand, GP utilizes the whole-genomic information to estimate breeding values (BVs) and predict individuals´ performance for targeted traits (Meuwissen et al. 2001). With genetic mapping approaches, such as Quantitative Trait Loci (QTL) analysis, previous studies on white spruce have uncovered the genetic architecture of growth (Beaulieu et al. 2011; Pelgas et al. 2011), wood quality and physical attributes (Beaulieu et al. 2011; Lamara et al. 2016), phenolic compounds (Laoué et al. 2021) and phenology (Pelgas et al. 2011). Additionally, GP studies in spruce populations, have explored the predictive capacity of genomic information for various traits across progeny test sites (Beaulieu et al. 2014; Gamal El-Dien et al. 2015, 2016; Hu et al. 2023; Nadeau et al. 2023). These studies highlight that the intricate genetic architecture of a trait can be further complicated by a strong genotype-by-environment interaction (G × E) (Raymond and Namkoong 1990; Muir et al. 1992; Li et al. 2017; Berlin et al. 2019).

Given the significant impact of G × E on the differential genomic adaptation in spruce (Depardieu et al. 2021), it becomes imperative to examine the genetic associations across different environments to better understand how specific SNPs affect trait variability under varying environmental conditions. This examination is also essential for identifying reliable genetic variants that demonstrate stable marker effects across diverse environments (El-Kassaby et al. 2024), which could help ensure accurate predictions of individual performance in target environments. However, with few exceptions, GWAS and GP of BVs have received little attention with regards to G × E, despite their importance to commercially important forest trees, such as white spruce. In the presence of significant G × E interactions, single-site GWAS analyses were independently carried out for each environment (Ghosh Dasgupta et al. 2021). While many studies in crop plants have explored G × E interactions using QTL analysis (e.g., Manneh et al. 2007; Ma et al. 2009), GWAS analyses specifically addressing G × E interactions are still rare (Comadran et al. 2011; Xu et al. 2014; Eltaher et al. 2021). In forest trees, few studies have examined G × E interaction using QTL analysis (Rae et al. 2008; Pelgas et al. 2011; Freeman et al. 2013) or used a candidate gene approach (Li et al. 2016) to assess SNP effects across different environments/sites. To our knowledge, this is the first study to investigate G × E interactions using GWAS in white spruce. Except for a few cases, GP of BVs have not been taken into account when modeling G × E in forest tree species, such as white spruce (Beaulieu et al. 2014; Gamal El-Dien et al. 2015; Hu et al. 2023; Nadeau et al. 2023).

Here, we conducted a comprehensive multi-environment GWAS and GP study for a total of 30 traits, including productivity, defense, and climate-adaptability traits in white spruce. Phenotypes were measured on three different sites in Alberta, Canada, providing a relatively broad range of climatic conditions (Rweyongeza et al. 2007). To achieve the research objectives, we analyzed 1540 white spruce trees representing a subset of open-pollinated progeny grown on three genetic (progeny) test sites across central Alberta (Thomas et al. 2019). Genotyping was performed using 467,224 SNP markers. Given the ecological and economic importance of white spruce, understanding the quantitative genetic underpinnings of trait variability across environmental variation is essential. Our findings provide a unique opportunity to evaluate the genetic architecture and G × E interaction for a suite of traits key to productivity, growth, and resilience to biotic and abiotic stresses, with anticipated significant implications for sustainable forest management practices, affecting future tree breeding decisions, particularly in response to rapidly changing climatic conditions.

## Materials and methods

### Genetic material and trial description

Genetic material and trials were previously described in (Cappa et al. 2022). Briefly, the genetic material comes from three open-pollinated white spruce progeny tests from Alberta Forestry and Park’s white spruce Region D1 controlled parentage program (FGRMS 2016). The entire population evaluated in the three progeny trials consisted of 150 families originating from 10 provenances. These trials were established on three sites in Alberta: Calling Lake (CALL, 55°16′N, 113°09′W, 640 m elevation), Carson Lake (CARS, 54°34′N, 115°34′W, 1006 m elevation), and Red Earth (REDE, 56°34′N, 115°19′W, 518 m elevation). The field experimental design for all locations was a randomized block design with six replicates and 5- or 6-tree row plots at 2.5 × 2.5 m spacing. All sites were fenced with a single border row of trees surrounding each trial.

### Traits evaluated

Two growth productivity traits, diameter at breast height (1.3 m; DBH) and tree height (HT) were measured at age-30. Wood density (WD) was assessed using 5 mm increment cores taken at DBH. Collected cores were Soxhlet extracted with acetone, cut to 1.68 mm thickness, and scanned by X-ray densitometry (Quintek Measurement Systems, TN) at a 0.0254 mm resolution. Average WD was calculated on an oven-dry weight basis, weighted by annual basal area increment (BAI), excluding rings before 1995. Microfibril angle (MFA) was determined by X-ray diffraction by determining the 002 diffraction arc (T-values) using a Bruker D8 Discover X-ray diffraction unit equipped with an area array detector (GADDS) on the radial face of individual growth rings (Ukrainetz et al. 2008). Additionally, two dendrochronological indices were generated from the individual tree ring data: drought resistance (Resistance) and mean drought sensitivity (Sensitivity). Resistance was calculated as the ratio of the BAI during the 2015 drought to the average BAI from the four preceding years (2011–2014), indicating how well a tree-maintained growth during drought. Sensitivity was calculated using the relative change in BAI between consecutive years to assess a tree’s responsiveness to climate fluctuations. Average stable carbon isotope ratio (δ^13^C) was also measured using wood core slabs retained during the pneumatic processing of density specimens. The slabs were dried, ground, and analyzed at Alberta InnoTech Stable Isotope Laboratory using a MAT 253 mass spectrometer with Conflo IV interface. Approximately 1 mg of ground sample was combusted to produce CO_2_, which was then analyzed by mass spectrometry for δ^13^C, with results normalized to Vienna Pee Dee Belemnite standards. These traits (DBH, HT, WD, MFA, Resistance, Sensitivity, δ^13^C) were previously analyzed in (Cappa et al. 2022), where further methodological details can be found.

Individual-tree secondary defense chemical compounds, including monoterpenes and polyphenolics, were identified and quantified from phloem tissue. Specifically, seven monoterpenes (α-pinene, β-pinene, camphene, myrcene, limonene, terpinolene, and camphor), including the sum of all hexane-extractable compound concentrations (total monoterpenes), were analyzed. The monoterpenes were also previously studied in (Cappa et al. 2022). In addition, 12 phenolics (gallic acid, gallocatechin, catechin, pungenol, caffeic acid, vanillin, taxifolin, quercetin, naringenin, kaempferol, apigenin, and isorhamnetin) were included in this study analyses.

Finally, for each tree, three gas exchange traits were assessed: stomatal conductance (gs), intrinsic water use efficiency (WUE), and intercellular CO_2_ concentration (Ci). WUE was calculated as the ratio of photosynthesis (A) to stomatal conductance (gs). We chose to focus on these gas exchange traits as they indicate a plants´ water usage strategy, and thus, potential adaptability to intensified droughts under climate change. The effects of vapor pressure deficit (VPD) and hour after sunrise (HAS) on the gas exchange traits were accounted for using a two-step analysis. Initially, we modeled the effect of VPD on each combination of trait-site and corrected for VPD’s influence individually. Following (Oren et al. 1999), as the responses of some of the gas exchange traits to VPD were not always linear, we employed the natural logarithm of VPD as a predictor for stomatal conductance at the CALL site and for all traits at the CARS site. Variation in trait responses to VPD across sites was likely influenced by the different sampling dates over the growing season, leading to disparities in both the VPD range and needle phenological stage between sites. Following VPD correction, we modeled the effect of HAS on each combination of VPD-adjusted trait value and site and accounted for the HAS effect. Given the correlation between VPD and HAS, we performed separate regressions of HAS against VPD, or the logarithm of VPD, for each trial and used the residual of HAS as the predictor (for details of the methods for measuring and correcting the gas exchange traits, see Wei et al. 2022). Supplementary Table S1 provides the list of traits, the total number of trees per trait, and summary statistics for all phenotypic traits in their original scale (i.e., before design adjustment and standardization).

Measurements were not available at all progeny test sites for all traits (see Supplementary Table S2). Twelve traits were assessed across three sites (HT, DBH, WD, MFA, Resistance, Sensitivity, δ¹³C, α-pinene, camphene, myrcene, limonene, total monoterpenes). However, eighteen traits were assessed across two sites: CALL and REDE (β-pinene, camphor, terpinolene, gallic acid, gallocatchin, catechin, pungenol, caffeic acid, vanillin, taxifolin, quercetin, naringenin, kaempferol, apigenin, isorhamnetin) and CALL and CARS (gs, WUE, and Ci).

To improve data normality prior to model fitting, a logarithmic transformation was applied to MFA, as well as all monoterpene and polyphenolic compounds, except for gallic acid, catechin, and vanillin (see Supplementary Fig. S1). Prior to GWAS and GP analyses, all phenotypic data were spatially adjusted (e.g., Dutkowski et al. 2016) using the design effects within a pedigree-based classical a *priori* design model for each trait, analyzed separately at each site. Design-adjusted phenotypic data for each trait and site were obtained by subtracting the estimated replication effects from the original phenotype for each tree (Cappa et al. 2022). The proportion of total variance accounted for by replication effects within each site for each trait is presented in Supplementary Table S2. Finally, all trait data were standardized to a mean of zero and a variance of one.

### Sample selection and genotyping

A subset of 80 families (out of 150), each with ≈eight individual trees per family for CALL and REDE and four individual trees per family for CARS, were selected based on tree height at age 30 (Cappa et al. 2022). To capture the extent of variability, this sub-sample was selected based on their low-, average-, and high- pedigree-based breeding values for height. An additional 142 potential forward selection trees, previously identified in the three progeny trials based on their high-height breeding values, were also included for sequencing. Among these 142 trees, 34 belonged to 19 additional open-pollinated families not included in the original 80 selected families. In summary, a total of 1625 trees from 99 open-pollinated families were measured, and their data analyzed.

DNA samples that passed the quality control requirements were genotyped using the genotyping-by-sequencing (GBS) platform (for details, see Chen et al. 2013). After filtering the SNP dataset for 30% missing data, a minor allele count of one, and a maximum, site read depth of 70 or less, a final set of 1599 trees and 467,224 (467K) biallelic SNPs was retained. Missing data were imputed using the average observed allele at each genetic locus (Ratcliffe et al. 2015).

### Pedigree correction

Using the filtered SNP subset, we examined the pairwise additive relationship coefficients of the ***G***-matrix to identify any discrepancies from the expected values. After removing 59 trees due to parent conflicts, manual assignment or reassignment of parentage was performed based on the observed deviations, such as those exceeding predefined thresholds for half-sibling relationships (e.g., 0.25). A final set of 1540 trees was used for subsequent analyses (for details, see Cappa et al. 2022).

### Genomic parameter estimation

To assess trait quantitative genetic characteristics, heritability and across-site genetic correlations, the following additive multi-environment individual-tree mixed model [1], was fitted for each trait:

1 $$\left[\begin{array}{c}\boldsymbol{y}_{1}\\ \boldsymbol{y}_{2}\\ \boldsymbol{y}_{3}\end{array}\right]=\left[\begin{array}{ccc}\boldsymbol{X}_{1} & 0 & 0\\ 0 & \boldsymbol{X}_{2} & 0\\ 0 & 0 & \boldsymbol{X}_{3}\end{array}\right]\left[\begin{array}{c}{\boldsymbol\beta }_{1}\\ {\boldsymbol\beta }_{2}\\ {\boldsymbol\beta }_{3}\end{array}\right]+\left[\begin{array}{ccc}\boldsymbol{Z}_{{a}_{1}} & 0 & 0\\ 0 & \boldsymbol{Z}_{{a}_{2}} & 0\\ 0 & 0 & \boldsymbol{Z}_{{a}_{3}}\end{array}\right]\left[\begin{array}{c}\boldsymbol{a}_{1}\\ \boldsymbol{a}_{2}\\ \boldsymbol{a}_{3}\end{array}\right]+\left[\begin{array}{c}\boldsymbol{e}_{1}\\ \boldsymbol{e}_{2}\\ \boldsymbol{e}_{3}\end{array}\right]$$

where, ${\boldsymbol{y}}=\left[{{\boldsymbol{y}}}_{1}^{{\prime} },{{\boldsymbol{y}}}_{2}^{{\prime} },{{\boldsymbol{y}}}_{3}^{{\prime} }\right]$ are the vector of individual tree adjusted-phenotypes for sites (1 = CALL, 2 = CARS, and 3 = REDE); ${\boldsymbol{\beta }}=\left[{{\boldsymbol{\beta }}}_{1}^{{\prime} },\,{{{\boldsymbol{\beta }}}_{2}^{{\prime} },\,{\boldsymbol{\beta }}}_{3}^{{\prime} }\right]$ is the vector of fixed effects of genetic group formed according to provenances; additive genetic effects (*i.e*., breeding values) random vector of ${\boldsymbol{a}}=\left[{{\boldsymbol{a}}}_{1}^{{\prime} },\,{{{\boldsymbol{a}}}_{2}^{{\prime} },\,{\boldsymbol{a}}}_{3}^{{\prime} }\right]$ is distributed as ${\boldsymbol{a}}{\boldsymbol{ \sim }}{\boldsymbol{N}}\left({\boldsymbol{0}},{{\boldsymbol{\Sigma }}}_{{\boldsymbol{a}}}{{\bigotimes }}{\boldsymbol{G}}\right)$, where **Σ**_***a***_ is the multi-environment genetic effects (co)variance matrix with dimension 3 × 3 and ***G***-matrix is the realized genomic relationship matrix (see below). Finally, ${\boldsymbol{e}}=\left[{{\boldsymbol{e}}}_{1}^{{\prime} },\,{{\boldsymbol{e}}}_{2}^{{\prime} },\,{{\boldsymbol{e}}}_{3}^{{\prime} }\right]$ is the random residual vector distributed as ${\boldsymbol{e}}{\boldsymbol{ \sim }}{\boldsymbol{N}}\left({\boldsymbol{0}},{{\boldsymbol{R}}}_{{\boldsymbol{0}}}{{\bigotimes }}{\boldsymbol{I}}\right)$, where ***R***_**0**_ is the residual (co)variance matrix for the three environments (sites) with dimension 3 × 3. We assumed an unstructured (co)variance matrix for the genetic effects (**Σ**_***a***_). However, given that the sites were assessed separately, the residual covariance across sites is assumed to be zero. The vector ***X***_1_, ***X***_2_ and ***X***_3_, and the matrices ${{\boldsymbol{Z}}}_{{a}_{1}}$, ${{\boldsymbol{Z}}}_{{a}_{2}}$ and ${{\boldsymbol{Z}}}_{{a}_{3}}$, relate the observation to the means of the site effects in ***β***, and the additive genetic effects for each tree in ***a***. The symbol “´”, indicates the transpose operation.

Following (VanRaden 2008), the genomic relationship matrix (***G***-matrix) based on 467K SNPs was calculated as follows:

$${\boldsymbol{G}}=\frac{{\bf{WW}}^{\boldsymbol{{\prime} }}}{2\sum {p}_{i}(1-{p}_{i})}$$

where, **W** is the *n* × *m* (*n* = number of individuals, *m* = number of SNPs) rescaled genotype matrix following **M** - **P**, where **M** is the genotype matrix containing genotypes coded as 0, 1, and 2 according to the number of alternative alleles, and **P** is a vector of twice the allelic frequency, *p*_*i*_.

The heat map of pair-wise genomic relationship (Supplementary Fig. S2) shows negligible population structure across the 10 provenances but clear family structure, with small squares along the diagonal representing groups of approximately eight, four, and eight trees for CALL, CARS, and REDE, respectively (Supplementary Fig. S2). Off-diagonal squares indicate related trees across sites. Additionally, histograms of the diagonal (Supplementary Fig. S3a) and off-diagonal (Supplementary Fig. S3b) elements of the ***G***-matrix depict the distributions of these coefficients.

Individual narrow-sense heritability (${\widehat{h}}^{2}$) and genetic correlations between sites (${\hat{r}}_{{a}_{{ij}}}$) were estimated as follows:

$${\widehat{h}}^{2}=\frac{{\widehat{\sigma }}_{a}^{2}}{{\widehat{\sigma }}_{a}^{2}+{\widehat{\sigma }}_{e}^{2}}{;\hat{r}}_{{a}_{{ij}}}=\frac{{\widehat{\sigma }}_{{a}_{i,j}}}{\sqrt{{\widehat{\sigma }}_{{a}_{i,i}}^{2}\times {\widehat{\sigma }}_{{a}_{j,j}}^{2}}}$$

where, ${\widehat{{\rm{\sigma }}}}_{{\boldsymbol{a}}}^{2}$ is the estimated variance for the additive genetic effects, and ${\widehat{{\rm{\sigma }}}}_{{\boldsymbol{e}}}^{2}$ is the estimated residual error.

Breeding values, variance components, and genetic correlations between sites from model [1] (and their standard errors) were estimated using average information restricted maximum likelihood (AI-REML) with the airemlf90 software, part of the blupf90+ suite (Lourenco et al. 2022) from the BLUPF90 family of programs (Misztal et al. 2018).

### Multi-environment GWAS analysis

For all traits, multi-environment GWAS was performed to obtain estimates of marker effects and their associated *p*-values from breeding values (Aguilar et al. 2019) estimated using the multi-environment individual-tree model [1] and the program postGSf90, which is also part of the BLUPF90 family (Misztal et al. 2018). The *p*-value for each *k* SNP from each individual trait using the multi-environment model [1] was computed using the following (Aguilar et al. 2019) formula:

$${p{{\_{\rm{value}}}}}_{k}=2\left(1-\Phi \left(\frac{{\widehat{g}}_{k}}{{sd}\left({\widehat{g}}_{k}\right)}\right)\right)$$

where, ${sd}\left({\widehat{g}}_{k}\right)$ is the standard deviation of the SNP effect estimate (${\widehat{g}}_{k}$) (${sd}\left({\widehat{g}}_{k}\right)=\sqrt{{Var}\left({\widehat{g}}_{k}\right)}$), ${Var}\left({\widehat{g}}_{k}\right)$ is the variance of the estimated SNP effects, and Ф(.) is the cumulative density function of the normal distribution. *P*-values are obtained by back solving for SNP effects based on the breeding value predictions. Positive associations were determined at the nominal *p*-value < 0.05 level, and a Bonferroni correction was used to control the family-wise error rate (FWER) (Stevens et al. 2017). It is important to note that associations were determined at the individual SNP level, which does not account for linkage disequilibrium (LD) between SNPs (Dehman et al. 2015). Therefore, we selected a -logP value of 6.97, derived by dividing the *p*-value = 0.05 by the total number of testing SNP markers in the analysis N = 467,224 (i.e., *p*-values of 1.07 ×10^−07^). Two arbitrary *p*-value thresholds of 1.07 ×10^−06^ and 1.07 ×10^−05^ were used for suggestive associations. Although these thresholds might be arbitrary, and some false positives may arise using this approach, it helps to compare the GWAS results across environments and traits.

### Multi-environment GP analysis

The multi-environment GP analysis utilized genomic variance components obtained from the multi-environment model [1] and involved all available trees with phenotype for each trait-site combination. A ten-fold cross-validation analysis was conducted across the 30 traits, where sampling was performed at individual tree level. In each fold, one subsample was used as the validation set, while the remaining nine subsamples were used as training. All trees with phenotypic data were in the training population at least once in each fold. Predictive ability was estimated by evaluating the Pearson correlation coefficient between the predicted breeding values from the full data set (i.e., using all the available phenotyped trees for each trait) and those from the validation set, adjusted by the square root of the narrow-sense heritability of each trait-site combination (Legarra et al. 2008). Prediction bias was calculated by regressing the observed tree breeding values from the full data set against those predicted from the validation set, with a regression coefficient of one indicating no bias. Differences in predictive ability and bias across sites were analyzed using an analysis of variance (ANOVA), followed by Tukey’s multiple comparison test, employed at a significance level α = 0.05. Cross-validation analyses were performed using the BLUPF90 family programs (Misztal et al. 2018) and automated with a customized R-script for each trait.

## Results

### Heritability estimates and correlations between sites

Based on the multi-environment GBLUP model [1], narrow-sense heritability estimates, when averaged across test sites, ranged from 0.20 to 0.94 (Table 1). Across traits, heritability estimates were highest at REDE (0.59), followed by CALL (0.53), while CARS exhibited moderate estimates (0.36) (Table 1). Across test sites, heritability estimates for growth traits, HT and DBH, showed high values across all three sites, ranging from 0.73 to 0.99, with averages of 0.94 and 0.55, respectively (the DBH at CARS site was the exception with a heritability estimate of 0.05). Narrow-sense heritability for wood quality traits varied, with values ranging from 0.19 to 0.82, with an average of 0.69 for WD and 0.31 for MFA. Both dendrochronological drought indices, Resistance and Sensitivity, showed moderate to high heritability estimates for CALL and REDE, with values ranging from 0.31 to 0.81 and averages of 0.38 and 0.47, respectively. However, these values were considerably lower at CARS (0.17 and 0.07, respectively). High heritability values were also found for δ¹³C for all sites. Monoterpene content, such as myrcene, camphene, and limonene, showed the highest heritability values, ranging from 0.15 to 0.96, with averages of 0.72, 0.59, and 0.55, respectively. β-pinene produced the lowest heritability values, averaging 0.33 across all three sites. Heritability estimates for phenolic traits varied from low (0.19) to high (0.89) across different compounds, with the highest value for caffeic acid (average 0.70) and the lowest value for kaempferol (average 0.25). Heritability estimates for gas exchange traits varied from low (0.15) to moderate (0.30), with averages of 0.20 (gs), 0.29 (WUE), and 0.25 (Ci). These findings provide valuable insights into the genetic basis of the assessed traits on different sites, emphasizing the potential for selective breeding to enhance productivity and adaptability in white spruce.

**Table 1:** Estimated genomic narrow-sense heritability (and approximate standard errors) for each of the 30 traits assessed in the white spruce population at three progeny test sites, CALL, CARS and REDE.

Trait/site	CALL	CARS	REDE
HT	0.99 (0.00)	0.86 (0.06)	0.98 (0.01)
DBH	0.73 (0.12)	0.05 (0.02)	0.87 (0.07)
WD	0.55 (0.08)	0.69 (0.11)	0.82 (0.09)
MFA^a^	0.44 (0.09)	0.29 (0.08)	0.19 (0.01)
Resistance	0.31 (0.07)	0.17 (0.03)	0.65 (0.08)
Sensitivity	0.54 (0.05)	0.07 (0.02)	0.81 (0.04)
δ¹³C	0.66 (0.17)	0.92 (0.28)	0.96 (0.19)
α-pinene^a^	0.62 (0.06)	0.10 (0.02)	0.52 (0.07)
β-pinene^a^	0.36 (0.07)	*b*	0.31 (0.04)
camphene^a^	0.96 (0.01)	0.15 (0.03)	0.66 (0.07)
camphor^a^	0.37 (0.08)	*b*	0.61 (0.08)
myrcene^a^	0.95 (0.23)	0.69 (0.30)	0.53 (0.17)
limonene^a^	0.76 (0.05)	0.51 (0.05)	0.38 (0.03)
terpinolene^a^	0.64 (0.08)	*b*	0.33 (0.04)
total monoterpenes^a^	0.66 (0.09)	0.34 (0.07)	0.68 (0.09)
gallic acid	0.50 (0.23)	*b*	0.45 (0.19)
gallocatchin^a^	0.36 (0.24)	*b*	0.75 (0.20)
catechin	0.25 (0.04)	*b*	0.78 (0.06)
pungenol^a^	0.19 (0.05)	*b*	0.51 (0.11)
caffeic acid^a^	0.89 (0.02)	*b*	0.51 (0.04)
vanillin	0.58 (0.03)	*b*	0.72 (0.02)
taxifolin^a^	0.29 (0.04)	*b*	0.50 (0.05)
quercetin^a^	0.22 (0.02)	*b*	0.71 (0.03)
naringenin^a^	0.69 (0.03)	*b*	0.50 (0.03)
kaempferol^a^	0.31 (0.05)	*b*	0.20 (0.03)
apigenin^a^	0.76 (0.02)	*b*	0.40 (0.03)
isorhamnetin^a^	0.38 (0.03)	*b*	0.65 (0.03)
gs	0.25 (0.06)	0.15 (0.04)	*b*
WUE	0.30 (0.04)	0.27 (0.04)	*b*
Ci	0.30 (0.04)	0.21 (0.03)	*b*

Abbreviations used for the traits are described in the text.

^a^Logarithmic transformed.

^b^Heritability estimates and their approximate standard errors were not calculated due to the unavailability or insufficiency of phenotypic data.

Across all traits, genetic correlations between sites ranged from 0.23 (Ci) to 1.00 (limonene). Average genetic correlation estimates were higher between CALL and REDE (average: 0.81, range: 0.44–0.99), and lower between CALL and CARS (average: 0.64, range: 0.03–0.98) and between CARS and REDE (average: 0.71, range: 0.50–1.00) (Table 2 and Supplementary Fig. S4). With few exceptions, productivity-related, adaptability, and defense-related monoterpenes and phenolic traits showed moderate (0.40 < between sites > 0.70) to high (>0.70) values of genetic correlation between sites. In contrast, genetic correlations for gas exchange traits (WUE and Ci) were low (<0.40). For productivity-related traits, HT and DBH showed a moderate to high degree of genetic correlation, suggesting a shared genetic influence across sites, except for HT between CALL and CARS (0.27). For wood quality traits, genetic correlations among sites were moderate to high, similarly for the adaptability-related drought indices, Resistance and Sensitivity, except for Resistance between CALL and CARS (0.05). Significant positive genetic correlations were found across sites for isotopic δ¹³C values (average: 0.97, range: 0.94–0.99). Traits related to defense mechanisms, such as α-pinene, myrcene, camphene, and limonene, consistently exhibited high genetic correlations between sites (>0.70), indicating a low level of G × E. However, phenolic traits, such as gallic acid, gallocatechin, vanillin, taxifolin, naringenin, apigenin, and isorhamnetin exhibited varying degrees of G × E, as evidenced by their genetic correlations (Table 2 and Fig. S4). Finally, together with the low to moderate genetic correlations observed for WUE, Ci, and gs (Table 2 and Supplementary Fig. S4), our results emphasize the importance of G × E when considering both abiotic (water stress) and biotic defense-related traits.

**Table 2:** Estimated genetic correlations (and approximate standard errors) between the three progeny sites for each of the 30 traits assessed in the white spruce population.

Traits	CALL-CARS	CALL-REDE	CARS-REDE
HT	0.27 (0.15)	0.85 (0.05)	0.52 (0.13)
DBH	0.40 (0.41)	0.90 (0.12)	0.72 (0.25)
WD	0.98 (0.07)	0.99 (0.06)	0.99 (0.04)
MFA^a^	0.53 (0.36)	0.95 (0.23)	0.76 (0.28)
Resistance	0.03 (0.17)	0.55 (0.15)	0.85 (0.05)
Sensitivity	0.83 (0.05)	0.98 (0.01)	0.69 (0.08)
δ¹³C	0.96 (1.60)	0.99 (1.18)	0.94 (1.38)
α-pinene^a^	0.98 (0.01)	0.67 (0.08)	0.50 (0.11)
β-pinene^a^	*b*	0.60 (0.12)	*b*
camphene^a^	0.63 (0.09)	0.60 (0.09)	0.90 (0.03)
camphor^a^	*b*	0.44 (0.10)	*b*
myrcene^a^	0.93 (2.39)	0.91 (3.73)	0.87 (1.43)
limonene^a^	0.99 (0.20)	1.00 (0.15)	1.00 (0.22)
terpinolene^a^	*b*	0.25 (0.10)	*b*
total monoterpenes^a^	0.93 (0.07)	0.98 (0.02)	0.93 (0.07)
gallic acid	*b*	0.51 (6.51)	*b*
gallocatchin^a^	*b*	0.63 (2.08)	*b*
catechin	*b*	0.91 (0.02)	*b*
pungenol^a^	*b*	0.92 (0.12)	*b*
caffeic acid^a^	*b*	0.82 (0.02)	*b*
vanillin	*b*	0.45 (0.04)	*b*
taxifolin^a^	*b*	0.67 (0.06)	*b*
quercetin^a^	*b*	0.39 (0.05)	*b*
naringenin^a^	*b*	0.51 (0.04)	*b*
kaempferol^a^	*b*	0.70 (0.08)	*b*
apigenin^a^	*b*	0.51 (0.04)	*b*
isorhamnetin^a^	*b*	0.47 (0.05)	*b*
gs	0.64 (0.16)	*b*	*b*
WUE	0.24 (0.09)	*b*	*b*
Ci	0.23 (0.10)	*b*	*b*

Abbreviations used for the traits and sites are described in the text.

^a^Logarithmic transformed.

^b^Correlations and their approximate standard errors were not estimated with the CARS and REDE sites due to the unavailability or insufficiency of phenotypic data.

In this study, we used genetic correlations among environments as an indicator of G × E interactions, treating a trait in two environments as distinct traits (Falconer and Mackey 1996). However, as demonstrated by (Fernando et al. 1984), estimates of genetic covariances can be biased in unbalanced data unless genetic and residual variances are identical across all sites. Supplementary Table S3 provides the estimates of additive genetic and residual variances for each of the 30 traits assessed in the studied white spruce population across three progeny test sites. Additionally, this table presents the G × E variance for each trait averaged across the three sites, calculated following (Itoh and Yamada 1990) (Equation 27). We also report the averaged G × E variance as a percentage of the total phenotypic variance.

### Genome‑wide association (GWAS) analysis

**Fig. 1:**
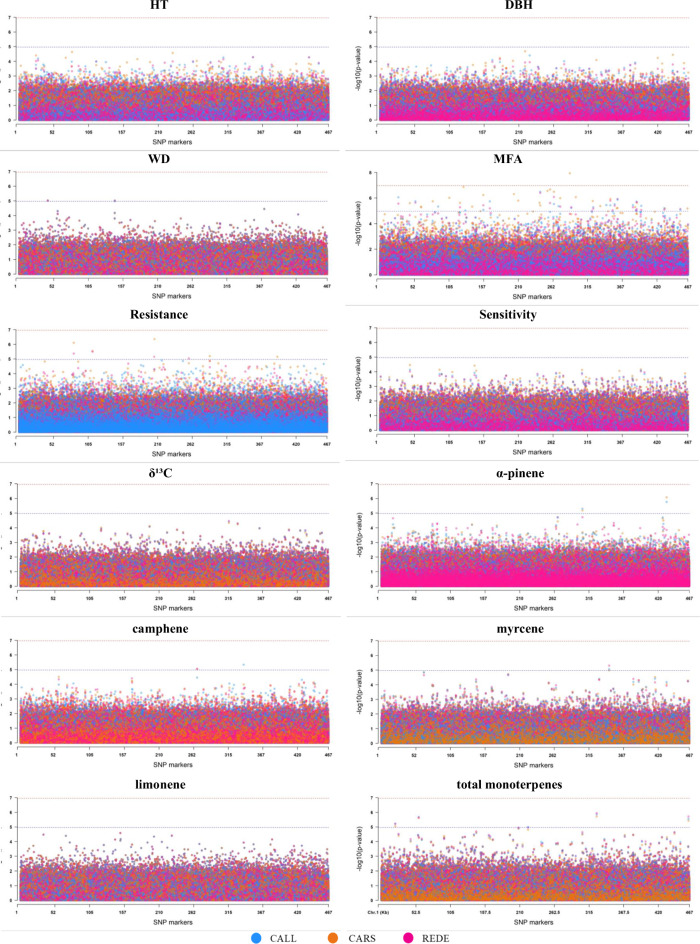
Manhattan plots for the multi-environment genome-wide association (GWAS) analyses for each of the 12 traits studied across the three sites (CALL, CARS, and REDE) in the white spruce population. The vertical *y*-axis indicates -log10(*p*-value) and the horizontal *x*-axis indicates the single-SNPs expressed in thousands of SNPs. The red dashed line represents the cutoff *p*-values of 1.07 ×10^−07^ based on adjusted Bonferroni correction ( -log10(*p*-value) equal to 6.97) and the blue dashed line represents the cutoff *p*-values of 1.07 ×10^−05^ (; -log10(*p*-value) equal to 4.97). Abbreviations used for the traits and sites are described in the text.

A total of 33,640,128 association tests were conducted, involving 467,244 SNPs across 30 traits and sites. The *p*-values were obtained from the breeding value predictions from the multi-environment individual-tree model [1]. Overall, among the 30 traits assessed across three and two sites, CALL showed the highest number of significant associations for the three critical values studied, with 287 significant associations, followed by REDE (279) and CARS (225). Specifically, the multi-environment GWAS with 467,244 SNPs for each of the 30 traits identified a total of 27, 8, and 27 SNPs, for CALL, CARS, and REDE, respectively. All these SNPs passed the Bonferroni correction *p*-value cutoff of 1.07 ×10^−07^ (-log10(*p*-value) = 6.97) (Table 3, Fig. 1 and Supplementary Fig. S5). With a slightly less stringent *p*-value of 1.07 ×10^−05^ (-log10(*p*-value) = 4.97), a total of 201, 174, and 188 significant SNPs were identified for CALL, CARS, and REDE, respectively (Table 3, Fig. 1 and Supplementary Fig. S5). At this *p*-value cutoff, approximately 20% of the significant associations were found with WUE, and 17.7% with Ci (Table 3 and Supplementary Fig. S5). Defense-related phenolic compounds, such as naringenin, accounted for 18.8% of the significant markers, while MFA contributed 11.1%. The remaining traits represent less than 5.7% of the significant associations.

**Table 3:** Number of strong and suggestive significant single-SNP association by trait and progeny test sites (CALL, CARS, REDE) from the three different *p*-values cutoff studied.

Traits	CALL			CARS			REDE		
	1.07 ×10^−7^	1.07 ×10^−6^	1.07 ×10^−5^	1.07 ×10^−7^	1.07 ×10^−6^	1.07 ×10^−5^	1.07 ×10^−7^	1.07 ×10^−6^	1.07 ×10^−5^
HT	0	0	0	0	0	0	0	0	0
DBH	0	0	0	0	0	0	0	0	0
WD	0	0	2	0	0	2	0	0	2
MFA^a^	0	2	23	2	11	31	0	2	25
Resistance	0	0	0	0	2	5	0	0	5
Sensitivity	0	0	0	0	0	0	0	0	0
δ¹³C	0	0	0	0	0	0	0	0	0
α-pinene^a^	0	0	2	0	1	2	0	0	0
β-pinene^a^	1	1	2	–	–	–	1	2	5
camphene^a^	0	0	1	0	0	1	0	0	1
camphor^a^	0	4	6	–	–	–	0	0	3
myrcene^a^	0	0	1	0	0	1	0	0	1
limonene^a^	0	0	0	0	0	0	0	0	0
terpinolene^a^	0	0	0	–	–	–	0	0	2
total monoterpenes^a^	0	0	5	0	0	4	0	0	4
gallic acid	0	1	2	–	–	–	1	3	17
gallocatchin^a^	0	0	1	–	–	–	0	0	0
catechin	0	0	1	–	–	–	0	0	1
pungenol^a^	2	8	11	–	–	–	2	8	12
caffeic acid^a^	4	4	7	–	–	–	3	6	10
vanillin	9	10	13	–	–	–	3	9	22
taxifolin^a^	0	0	8	–	–	–	0	0	0
quercetin^a^	0	0	3	–	–	–	10	0	1
naringenin^a^	7	11	34	–	–	–	0	24	64
kaempferol^a^	2	7	10	–	–	–	7	9	10
apigenin^a^	0	1	2	–	–	–	0	1	3
isorhamnetin^a^	0	0	1	–	–	–	0	0	0
gs	0	1	10	0	1	10	–	–	–
WUE	1	5	26	4	16	66	–	–	–
Ci	1	4	30	2	12	52	–	–	–
**Total**	27	59	201	8	43	174	27	64	188

Abbreviations used for the traits and sites are described in the text.

**Fig. 2:**
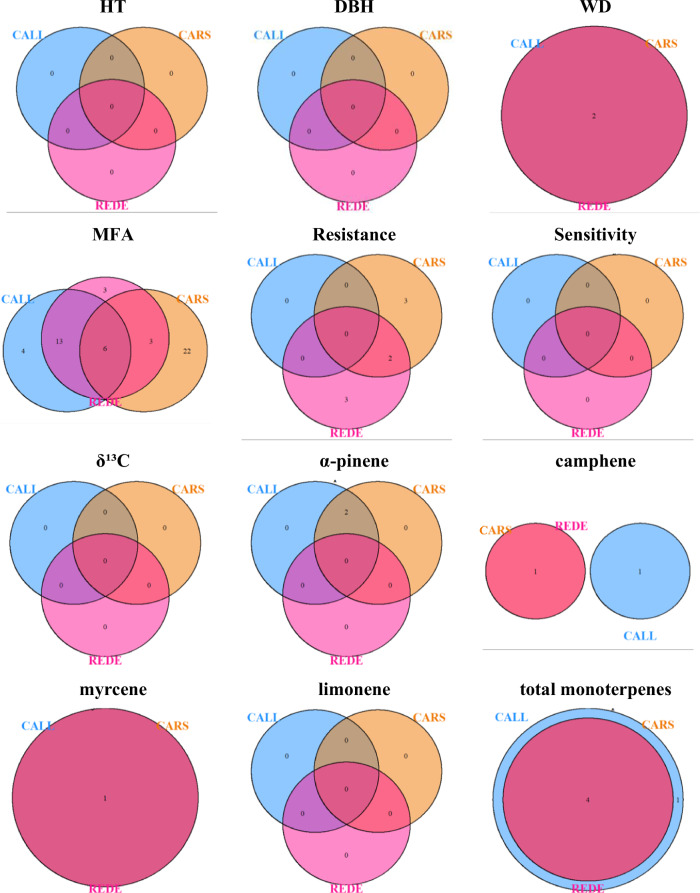
Venn diagram illustrating the overlap of significant genetic associations (suggestive cutoff *p*-values of 1.07 ×10^−05^) across the three different sites (CALL, CARS, and REDE). The numbers within the diagram indicate the count of SNPs significantly associated with the trait in each environment. Overlapping regions show the number of SNPs that are commonly associated across all three environments, highlighting the consistency or variability of genetic effects. Abbreviations used for the sites and traits are described in the text.

The shared and unique significant associations (suggestive cutoff *p*-values of 1.07 ×10^−05^) were visualized using Venn diagrams for all the 30 traits assessed across the three (CALL, CARS and REDE; Fig. 2) or two sites (CALL and REDE or CALL and CARS; Supplementary Fig. S6). Out of 563 significant SNPs across all traits and sites (Table 3), 105 showed significant associations at more than one site. Among these, more than 50% of genetic associations of WD, myrcene, total monoterpenes, α-pinene, and catechin are shared across environments. In contrast, Ci and WUE demonstrated greater site-specific genetic association, with only 1.2 and 1.1% of the associated variants shared between sites, respectively.

The QQ plot compares the observed distribution of *p*-values to the expected distribution under the null hypothesis. To assess the presence of significant genetic associations for the studied traits across environments (sites), QQ plots were generated for each environment and each of the 30 traits analyzed across three or two sites (Supplementary Fig. S7). For some traits, e.g., MFA at CARS and gallic acid at REDE, the QQ plots for different sites revealed distinctive deviations from the null hypothesis, showing pronounced tails above the expected line, and indicating potentially a higher number of associations than expected for a given site.

Our results revealed a robust positive correlation between Spearman rank correlations between the *p*-value for different pairs of sites and genetic correlations from the GBLUP model (R^2^ = 0.91; Supplementary Fig. S8). When the genetic correlation between sites was above 0.70, the Spearman rank correlation between *p*-values ranged from 0.76 to 1.00, with smaller Spearman rank correlations tending to correspond to a higher G × E interaction (lower genetic correlation). However, when the genetic correlation was below 0.70, the Spearman rank correlation between *p*-value ranged from 0.14 to 0.95.

We further assessed the variation in the number of significant SNP associations among the studied traits, revealing intriguing patterns in their distribution. Gas exchange traits (Ci and WUE), although assessed in only two of the three studied environments, exhibited the highest number of significant SNP associations (82 and 92, respectively; Table 3 and Supplementary Fig. S5). In contrast, growth traits (HT and DBH), dendrochronological indices (Sensitivity and Resistance), and limonene defense-monoterpene traits (Table 3 and Fig. 1), all of which were assessed at the three sites, showed no associations at the studied *p*-value thresholds. This absence of SNP associations suggests a complex interplay of genetic factors, indicative of a complex trait architecture following the infinitesimal model.

Due to the lack of a chromosome-level reference assembly, it is challenging to fully capture the linkage disequilibrium (LD) structure for these white spruce populations. However, when examining the interdependence of SNPs of the 363 unique significantly associated variants at a *p*-value threshold of 1.07 ×10^−5^, our analysis indicates that only 0.07% of the associated variants are in a LD > 0.90 (45 out of 65,703 SNP pairs), suggesting a low level of LD among most SNPs as a result of rapid LD decay in conifer genomes (Pavy et al. 2012).

### Genomic prediction (GP) analysis

**Fig. 3:**
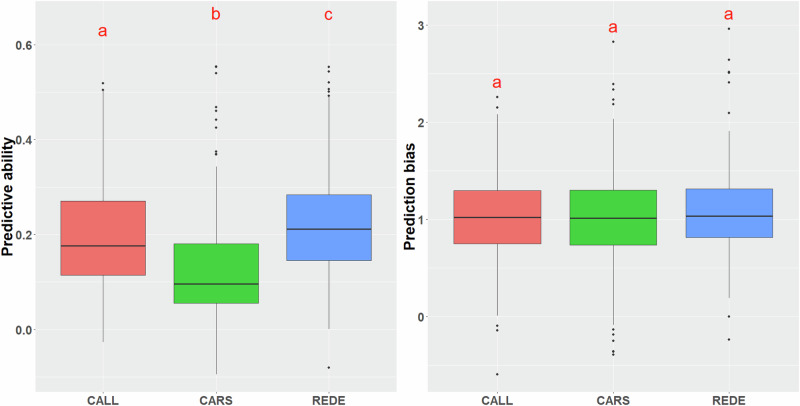
Average predictive ability and prediction bias across the 30 traits studied for the three sites (CALL, CARS, and REDE). Common letters above box-plots are not significantly different (α = 0.05). Abbreviations used for the sites are described in the text.

From the Tukey’s test (α = 0.05), the average predictive ability (PA) across the 30 studied traits differed significantly among the three sites. Specifically, REDE had the highest PA value at 0.22, followed by CALL (0.20) and CARS (0.13) (Fig. 3, Supplementary Table S4). However, the average prediction bias (PB) across traits did not show significant differences among sites (Fig. 3, Supplementary Table S4).

**Fig. 4:**
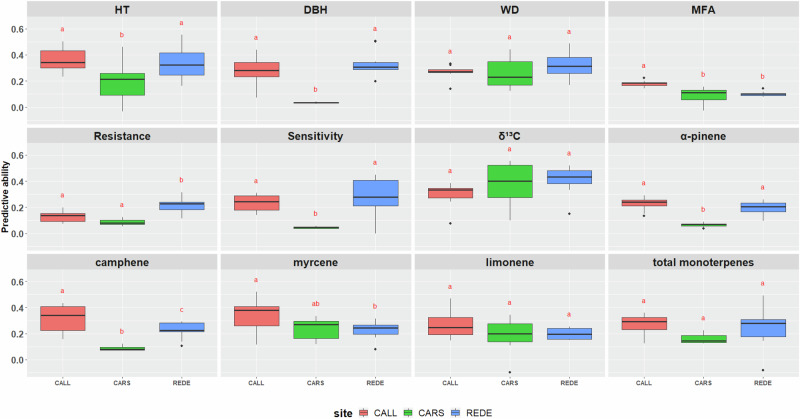
Average predictive ability for each of the 12 traits studied across the three sites (CALL, CARS, and REDE). Common letters above box-plots are not significantly different (α = 0.05). Abbreviations used for the traits and sites are described in the text.

**Fig. 5:**
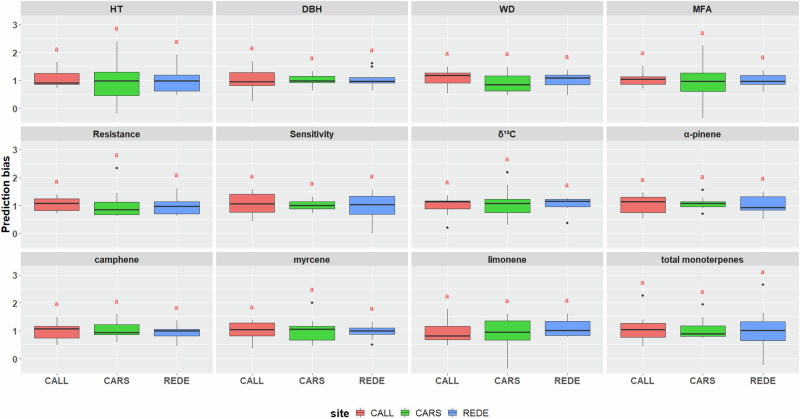
Average prediction bias for each of the 12 traits studied across the three sites (CALL, CARS, and REDE). Common letters above box-plots are not significantly different (α = 0.05). Abbreviations used for the traits and sites are described in the text.

Based on all traits studied, 20 exhibited significant differences among sites where they were assessed. Specifically, these included the 8 out of 12 traits assessed at three sites (CALL, CARS, and REDE) (Fig. 4, Supplementary Table S4) and 12 out of 18 traits assessed at two sites (CALL and REDE or CALL and CARS) (Supplementary Fig. S9, Supplementary Table S4). These findings indicate variation in PA across sites, emphasizing the influence of environmental factors. Nonetheless, it is important to note that only two traits (kaempferol and gs) demonstrated statistical differences in PB (Supplementary Fig. S10, Supplementary Table S4). In comparison, the remaining 28 traits were consistent across sites, suggesting an unbiased performance in trait prediction (Fig. 5, Supplementary Fig. S10, and Supplementary Table S4).

In summary, PA ranged from 0.03 to 0.41 across all trait-environment (site) combinations. For productivity-related traits, HT exhibited the highest PA at CALL (0.35), followed by REDE (0.34) and CARS (0.19), whereas the PA for DBH was significantly lower at CARS (0.03) compared to CALL (0.28) and REDE (0.33). For wood quality traits, WD ranges from 0.32 (REDE) to 0.19 (CARS), averaging 0.30, while MFA displayed a lower PA average of 0.12, ranging from 0.18 (CALL) to 0.09 (CARS). The adaptability-related drought indices, Resistance and Sensitivity, both exhibited moderate to low PA values with an average of 0.14 and 0.19, respectively, with CARS showing the lowest PA values for both traits (0.09 and 0.05, respectively). The stable carbon isotope ratio (δ^13^C), however, had a high PA of 0.36, with values ranging from 0.30 (CALL) to 0.41 (REDE). For defense-related monoterpene traits, averaged across sites, the PA values ranged from 0.12 (β-pinene) to 0.27 (myrcene) (Fig. 4, Supplementary Fig. S9, and Supplementary Table S4). The lowest PA for these monoterpenes was found in CARS. Similarly, the defense-related phenolic traits displayed PA values ranging from 0.13 (pungenol) to 0.30 (caffeic acid). Finally, gs, WUE, and Ci displayed low PA values of 0.06, 0.08, and 0.07, respectively (Supplementary Fig. S9, and Supplementary Table S4).

Among the 30 traits examined, as expected, a relatively high correlation was observed between PA and narrow-sense heritability (R^2^ = 0.64; see Supplementary Fig. S11). This correlation highlights the fact that genomic estimated breeding values derived from traits with high narrow-sense heritability offer greater PA compared to those from traits with lower narrow-sense heritability.

Regarding PB, the regression coefficients across the 30 traits examined in the three sites varied from 0.64 to 1.48 (Fig. 5, Supplementary Fig. S10, and Supplementary Table S4). However, a majority (80%) of the site-trait PB values fell within a narrow range of 0.90 to 1.10, suggesting a low level of bias across all traits and assessed sites. Notably, WUE exhibited the lowest PB value at CALL (0.64) and the second-to-last highest value at CARS (1.32) (Supplementary Fig. S10, and Supplementary Table S4). However, the remaining traits generally displayed no bias.

## Discussion

Exploring the intricate relationship between phenotype, genotype, and environment has become a critical research focus in quantitative genetics (Crossa et al. 2021). In this context, integrating GWAS and GP can enhance our understanding of the genetic architecture responsible for tree productivity and climate adaptability, while also facilitating the development of predictive models for estimating the performance of untested individuals in a population. Ultimately, these findings provide valuable insights for breeding programs aimed at producing improved genetic stock. We genotyped and dissected the genetic architecture of 1540 white spruce trees using 467,224 SNP markers across three sites that span a 500-meter elevation difference (Cappa et al. 2022). We examined 30 phenotypes including productivity, adaptability, defense monoterpenes and phenolics, and gas exchange traits assessed across three or two sites. Our multi-environment GWAS revealed several significant associations across traits and sites. While some genetic associations were shared across sites, indicative of common genetic determinants influencing trait variability in different environments, others were specific to particular sites. In the multi-environment GP analysis, predictive ability (PA) exhibited significant differences across sites, with the highest PA observed at REDE, a low elevation site characterized by a dry climate (Hogg et al. 2005; Cappa et al. 2022). However, prediction bias (PB) showed no significant differences.

### Genome‑wide association (GWAS) analysis

The importance of G × E interactions can be assessed by examining the genetic correlations among environments, treating a trait observed in two environments as two distinct traits (Falconer and Mackey 1996). This approach offers valuable insights into the extent of variation in the SNP associations among different sites. Access to genomic information has permitted exploiting G × E at the level of SNP × environment (site) interactions. As we show, low to moderate G × E interactions were prevalent for nearly all traits (Table 2 and Supplementary Fig. S4), except for the gas exchange traits (Ci and WUE) and terpinolene and quercetin, which exhibited low correlations (< 0.40; i.e., high G × E interactions).

Studies in crops (e.g., Comadran et al. 2011; Eltaher et al. 2021) and tree (Rae et al. 2008; Freeman et al. 2013) species have consistently shown that G × E can diminish the capacity to identify consistent QTLs across environments. This phenomenon is further elucidated in Supplementary Fig. S8, where traits with lower genetic correlation demonstrate inconsistent GWAS significance across sites. For instance, the gas exchange traits Ci and WUE, with a genetic correlation of 0.23 and 0.24, respectively (Table 2 and Supplementary Fig. S4), exhibited minimal SNPs overlapping across sites (1.2 and 1.1%, respectively; Supplementary Fig. S6). In contrast, traits with high genetic correlation, such as WD, total monoterpenes, and myrcene, demonstrated the greatest overlap among sites (66.7, 61.5, and 66.7%, respectively) (Fig. 2). These GWAS results reiterate the substantial influence of G × E within the Alberta Forestry and Park’s white spruce Region D1 program (FGRMS 2016), with over 80% of associated variants being site-specific (only 105 out of 563 associated markers across the 30 traits studied overlapped in two or more environments, Table 3). Similar findings have been reported for yield and sugar content in sugarcane (Wei et al. 2010). As a result, caution should be exercised when extrapolating association results to other environments (Mohammadi et al. 2020), and genetic markers identified for one site may not be applicable to different locations while considering marker-assisted selection (MAS) (Liu et al. 2006).

Our findings revealed substantial variation in genetic associations across different growing environments (Table 3, Figs. 1, and Supplementary Fig. S5). Notably, the CARS site, characterized by the highest elevation, highest mean annual temperature, lowest mean warmest month temperature (i.e., coolest summer), and the highest annual precipitation and moisture (see Table 1 in (Cappa et al. 2022), exhibited the lowest number of associated variants (174, *p*-value 1.07 ×10^−07^). This variation in associated SNP variants due to environmental differences align with findings from GWAS analyses in crops (Fikere et al. 2020; Tsai et al. 2020; López-Hernández et al. 2023). For instance, (López-Hernández et al. 2023) showed, in common bean across four sites spanning an environmental gradient in the Caribbean coast of Colombia, that the Caribia locality, which is located in the wet Caribbean subregion, was the one with the most associated markers, followed by the humid Turipaná and the drier Motilonia environments. Moreover, a discrepancy in white pine weevil (*Pissodes strobi* Peck) infestation rates among the studied sites may play a significant role in shaping the observed variations in genetic associations. As mentioned previously by Cappa et al. (2022), the sites CALL and REDE experienced infestations by the white pine weevil, a notorious pest known for its destructive impact on leading shoot growth. In contrast, CARS exhibited a notably lower incidence of weevil infestation. Reflecting on these findings, it presents an opportunity for future research to delve deeper into the influence of weevil infections on genetic associations.

The sample size utilized in a GWAS analyses plays a crucial role in determining the power to detect significant marker-trait associations (Uffelmann et al. 2021). For traits with smaller sample sizes across sites (i.e., those assessed across only two sites, see Supplementary Table S2), such as certain monoterpene and phenolic compounds, and gas exchange traits, the power to detect significant SNPs may be inherently reduced, resulting in fewer detected associations and less robust conclusions for these traits. In contrast, traits assessed at three sites with a larger sample size, such as growth, wood quality, dendrochronological indices, stable carbon isotope ratios, and certain monoterpene compounds, benefited from increased statistical power, yielding a higher number of significant marker-trait associations. Although sample size variation across sites within each trait could also affect the power of our analyses, the impact is mitigated by the multi-environment approach that accounts for correlations between environments and allows for “borrowing strength” across correlated sites during the analysis. This can be seen at the CALL and REDE sites, where larger sample sizes of 490 and 492 trees were assessed, on average per trait, respectively, enhancing the statistical power to identifying single-SNP associations, resulting in a higher number of both strong and suggestive associations (Table 3). Conversely, the CARS site, with a smaller sample size of 289 trees on average per trait, shows reduced capacity, suggesting that the “borrowing strength” of the multi-environment approach was still compensated.

It is important to note that while the single SNP-trait associations identified in this study provide valuable insights, we acknowledge that the number of detected SNPs may not be optimal. Studies have proposed simultaneously fitting all SNPs as random effects, to allow for the estimation of each SNP’s effect conditional on the effects of all other loci. This approach could effectively account for the underlying population genetic structure, as well as the LD among the analyzed SNPs. For example, (Kemper et al. 2012) demonstrated the benefits of treating marker effects as random and fitting all markers simultaneously to prevent spurious associations. This approach has been successfully applied to various animal (e.g., Hayes et al. 2010; Fan et al. 2011) and plant (e.g., Kristensen et al. 2018; Li et al. 2018) species studies. However, given the complexity of the genetic architecture underlying the phenotypes investigated in this study, it is unlikely that any single method can consistently achieve the same statistical power across different phenotypes (Ramzan et al. 2020).

### Genomic prediction (GP) analysis

Genomic prediction (GP) has been extensively explored in forest trees, yielding promising results across various productivity-related traits (Grattapaglia 2022; Isik 2022). However, the inherent presence of G × E can diminish the PA of predicted genomic breeding values (Hayes et al. 2009), owing to the diverse environmental conditions in tree improvement programs (Lebedev et al. 2020). In our study, even within the same breeding region (D1), the test sites experienced diverse climatic conditions (Cappa et al. 2022). This variability has contributed to the significant variation observed in the PA of the multi-environment prediction model across these sites (average across traits ranging from 0.13 to 0.22; see Fig. 3, Supplementary Table S4).

In white spruce Nadeau et al. (2023) reported variation in PA for wood and growth traits across two sites, with differences up to 29.03%. A similar scenario was observed in interior spruce (*Picea engelmannii* *×* *glauca*) (Gamal El-Dien et al. 2015), where G × E interactions impacted PA across sites for seven growth and wood quality traits analyzed. These finding are consistent with conclusions reached in spruce trees, where strong G × E for growth traits resulted in lower PAs across sites compared to the within-site cross-validation (Beaulieu et al. 2014; Chen et al. 2018). Collectively, these authors emphasized the necessity of incorporating G × E interactions into the GP model when significant G × E effects are detected.

Furthermore, our findings highlight a moderate to strong G × E interaction for growth traits and dendrochronological indices such as Resistance and Sensitivity (Table 2 and Supplementary Fig. S4), leading to notable differences in the PA across sites (Fig. 4). Conversely, WD, certain monoterpene compounds, and isotopic δ¹³C values showed relatively consistent PA values across sites due to lower G × E interaction (Figs. 4 and Supplementary Fig. S9). These observations align with similar findings reported by (Hu et al. 2023) in an interior spruce population, indicating that the impact of G × E on the PA may vary depending on the trait of interest (Lebedev et al. 2020). In particular, traits such as gas exchange may be particularly influenced by the specific environmental conditions of the year of phenotyping (e.g., Trenti et al. 2021), as these traits can respond rapidly to short-term climatic variations and stressors (McAusland et al. 2016; Lawson and Vialet-Chabrand 2019). In contrast, growth traits such as HT and DBH tend to integrate environmental effects over a longer period, making them less susceptible to annual fluctuations. Our results suggest that, especially for traits that are not subjective to G × E interactions, the genomic model developed and trained at a specific site could be effectively employed to predict genomic BVs at another site with comparable environmental conditions. However, for traits such as gas exchanges that exhibit greater sensitivity to G × E, accounting for these interactions is highly recommended.

The data presented in Table 1 showcases a range of narrow-sense heritability estimates derived using multi-environment GBLUP models (Model [1]). These results showed some differences in trait heritability across the three progeny test sites (CALL, CARS, and REDE), with particularly the low heritability estimates of CARS site. For example, the heritability of DBH and α-pinene at CARS was significantly lower (0.05 and 0.10, respectively) as compared to the other sites (e.g., 0.73 and 0.62 for CALL, 0.87 and 0.52 for REDE). This trend was consistent across several traits, including Resistance (0.17 for CARS vs. 0.31 for CALL and 0.65 at REDE) and Sensitivity (0.07 for CARS vs. 0.54 for CALL and 0.81 at REDE). The lower heritability estimates at CARS could be attributed to the environmental factors specific to this site (see Table 1 in (Cappa et al. 2022), which may have influenced the phenotypic expression of these traits, thereby reducing their genetic signal. Additionally, discrepancies in white pine weevil infestation rates among the studied sites, as previously mentioned, may have significantly shaped the observed variation in these estimates. These findings highlight the need for further research into the influence of site-specific environmental conditions, including weevil infestations, to better understand their impact on trait heritability and PA across different environments.

Moreover, these narrow-sense heritability estimates demonstrated a significant correlation with PA (R^2^ = 0.91, Supplementary Fig. S8), consistent with previous simulation-based studies (Meuwissen et al. 2001; Solberg et al. 2008; Jia and Jannink 2012), and empirical studies involving forest trees (Resende et al. 2012; Calleja-Rodriguez et al. 2020). This outcome is aligned with expectations, given that traits with higher heritability exhibit a close correspondence between observed phenotypic traits and underlying genetic values. In summary, the effectiveness of GP is closely linked to heritability, and genomic estimated breeding values are potentially more reliable for traits exhibiting high multi-environment heritability.

## Conclusion

We demonstrated that the integration of GWAS and GP techniques offers valuable insights into the complex interplay between genotype, phenotype, and environment in white spruce trees grown in central Alberta, Canada. Our study elucidated significant genetic associations across multiple environments, emphasizing the importance of considering G × E interactions in breeding programs. While some traits showed consistent genomic prediction across sites, others exhibited notable variability, highlighting the necessity of incorporating G × E interactions into predictive models for accurate estimation of performance across diverse environmental conditions. These findings contribute to the development of sustainable forest management strategies and will help inform the future breeding strategies for white spruce with enhanced productivity and adaptability, and ultimately ensure the long-term resilience and economic viability of white spruce resources.

## Supplementary information

Supplementary material

## Data Availability

Genotyping-by-sequencing (GBS) raw reads used in this study have been deposited in NCBI SRA BioProject - PRJNA748443 (https://www.ncbi.nlm.nih.gov/bioproject/PRJNA748443). Information of the white spruce trials including pedigree and adjusted and standardized phenotypic data are available in the GitHub repository: https://github.com/RESFOR/quantitative_genetics_R/blob/main/White_Spruce_Pedigree_Phenotype_Heredity2023.txt.
