# Correction: Revealing stable SNPs and genomic prediction insights across environments enhance breeding strategies of productivity, defense, and climate-adaptability traits in white spruce

**DOI:** 10.1038/s41437-025-00757-x

**Published:** 2025-03-24

**Authors:** Eduardo P. Cappa, Charles Chen, Jennifer G. Klutsch, Jaime Sebastian-Azcona, Blaise Ratcliffe, Xiaojing Wei, Letitia Da Ros, Yang Liu, Sudarshana Reddy Bhumireddy, Andy Benowicz, Shawn D. Mansfield, Nadir Erbilgin, Barb R. Thomas, Yousry A. El-Kassaby

**Affiliations:** 1 Instituto Nacional de Tecnología Agropecuaria (INTA), Instituto de Recursos Biológicos, Centro de Investigación en Recursos Naturales, De Los Reseros y Dr. Nicolás Repetto s/n, 1686 Hurlingham Buenos Aires, Argentina; 2 Consejo Nacional de Investigaciones Científicas y Técnicas (CONICET), Buenos Aires, Argentina; 3 Department of Biochemistry and Molecular Biology, Oklahoma State University, Stillwater, OK 74078 USA; 4 Department of Renewable Resources, University of Alberta, 442 Earth Sciences Bldg., Edmonton, AB T6G 2E3 Canada; 5 Department of Forest and Conservation Sciences, Faculty of Forestry, University of British Columbia, Vancouver, BC V6T 1Z4 Canada; 6 Department of Wood Science, Faculty of Forestry, University of British Columbia, Vancouver, BC V6T 1Z4 Canada; 7 Department of Biological Sciences, University of Alberta, P217 Biological Sciences Building, Edmonton, AB T6G 2E9 Canada; 8 Forest Stewardship and Trade Branch, Alberta Forestry and Parks, Edmonton, AB T6H 5T6 Canada; 9 Department of Botany, Faculty of Science, University of British Columbia, Vancouver, BC V6T 1Z4 Canada; 10 Present Address: Natural Resources Canada, Canadian Forest Service, Northern Forestry Centre, Edmonton, AB T6H 3S5 Canada; 11 Present Address: Irrigation and Crop Ecophysiology Group, Instituto de Recursos Naturales y Agrobiología de Sevilla, Avenida Reina Mercedes, 10, 41012 Sevilla, Spain; 12 Present Address: Department of Chemistry, University of Saskatchewan, Saskatoon, SK S7N 5E2 Canada

**Keywords:** Plant breeding, Genomics

Correction to: *Heredity* 10.1038/s41437-025-00747-z, published online 12 February 2025

The article “Revealing stable SNPs and genomic prediction insights across environments enhance breeding strategies of productivity, defense, and climate-adaptability traits in white spruce”, written by Eduardo P. Cappa, Charles Chen, Jennifer G. Klutsch et al., was originally published under exclusive license to “The Author(s), under exclusive licence to The Genetics Society”. As a result of the subsequent decision to publish the article under the open access model, the article’s copyright notice was changed on 03 March 2025 to © The Author(s) 2025 and the article is now distributed under a Creative Commons Attribution 4.0 International License, which permits use, sharing, adaptation, distribution and reproduction in any medium or format, as long as you give appropriate credit to the original author(s) and the source, provide a link to the Creative Commons licence, and indicate if changes were made. The images or other third party material in this article are included in the article’s Creative Commons licence, unless indicated otherwise in a credit line to the material. If material is not included in the article’s Creative Commons licence and your intended use is not permitted by statutory regulation or exceeds the permitted use, you will need to obtain permission directly from the copyright holder. To view a copy of this licence, visit http://creativecommons.org/licenses/by/4.0.

The original article has been corrected.

