# Supplementary material for: Revealing stable SNPs and genomic prediction insights across environments enhance breeding strategies of productivity, defense, and climate-adaptability traits in white spruce
Source: Heredity (Edinb). 2025 Feb 12;134(3-4):186–99. doi: 10.1038/s41437-025-00747-z (PMC11977214; doi:10.1038/s41437-025-00747-z)
Supplement: Supplementary file 1 — Supplementary material [file 41437_2025_747_MOESM1_ESM.pdf]

Table S1. Summary statistics for the 30 traits assessed in the white spruce population based on raw phenotypes. Abbreviations used for the traits are described in the text. Monoterpene concentrations are reported on a dry weight basis.

Type of trait	Trait full name	Trait	Unit	N	Mean	SD	CV	Min	Max
**Productivity**	**Tree height**	**HT**	cm	1516	947.32	171.96	0.18	200	1350
	**Diameter at breast height**	**DBH**	cm	1516	14.94	3.32	0.22	1.6	26
	**Wood density**	**WD**	$kg.m^{-3}$	1448	377.32	28.94	0.08	304.07	497.64
	**Microfibril angle**	**MFA**	°	1510	21.18	3.93	0.19	17.15	56.79
**Climate-adaptability**	**Drought resistance**	**Resistance**	-	1435	0.57	0.14	0.25	0.23	1.33
	**Mean drought sensitivity**	**Sensitivity**	-	1445	0.23	0.07	0.29	0.03	0.45
	**Average stable carbon isotope ratio**	**$\delta^{13}C$**	-	1509	-25.9	0.68	-0.03	-28.14	-23.55
**Defense chemical compounds**	**α-pinene**	**α-pinene**	$ng\ mg^{-1}$	1418	169.67	151.33	0.89	13.99	1502.32
	**β-pinene**	**β-pinene**	$ng\ mg^{-1}$	932	30.49	20.02	0.66	8.18	215.51
	**camphene**	**camphene**	$ng\ mg^{-1}$	1362	367.39	356.03	0.97	10.43	2585.76
	**camphor**	**camphor**	$ng\ mg^{-1}$	1183	758.19	677.58	0.89	17.79	5769.53
	**myrcene**	**myrcene**	$ng\ mg^{-1}$	1472	358.54	377.38	1.05	13.79	5644.61
	**limonene**	**limonene**	$ng\ mg^{-1}$	1472	429.13	425.42	0.99	10.9	3590.68
	**terpinolene**	**terpinolene**	$ng\ mg^{-1}$	906	39.36	22.76	0.58	8.17	169.09
	**total monoterpenes**	**total monoterpenes**	$ng\ mg^{-1}$	1495	2934.11	2425.41	0.83	13.1	18719.14
	**gallic acid**	**gallic acid**	mg/kg	947	0.67	0.17	0.26	0.02	1.55
	**gallocatechi**	**gallocatechi**	mg/kg	948	233.58	128.38	0.55	14.22	850.74
	**catechin**	**catechin**	mg/kg	948	3384.74	1201.01	0.35	408	8467
	**pungenol**	**pungenol**	mg/kg	948	82.37	51.88	0.63	10.63	460
	**caffeic acid**	**caffeic acid**	mg/kg	948	4.36	4.28	0.98	0.3	39.23
	**vanillin**	**vanillin**	mg/kg	948	12.05	4.74	0.39	2.88	28.4
	**taxifolin**	**taxifolin**	mg/kg	928	19.23	9.71	0.50	7	80.9
	**quercetin**	**quercetin**	mg/kg	916	8.53	10.11	1.18	1	70.6
	**naringenin**	**naringenin**	mg/kg	948	1.41	3.3	2.34	0.16	38.54
	**kaempferol**	**kaempferol**	mg/kg	948	58.42	82.56	1.41	1.33	507.22
	**apigenin**	**apigenin**	mg/kg	947	0.45	0.21	0.47	0.05	3.13
	**isorhamnetin**	**isorhamnetin**	mg/kg	948	0.64	0.72	1.12	0.1	6.08
**Climate-adaptability**	**Stomatal conductance**	**gs**	-	571	0.18	0.12	0.65	0.02	0.71
	**Intrinsic water use efficiency**	**WUE**	-	571	0.08	0.03	0.39	0.02	0.21
	**Intercellular CO_2 concentration**	**Ci**	-	571	247.09	47	0.19	54.01	359.04

NOTE: Number of trees for which trait values were used (N), mean, standard deviation (SD), phenotypic coefficient of variation (CV), minimum (Min.), and maximum (Max.) values observed.

Table S2. Number of trees sampled, and proportion of the total variance accounted for by the replication effects (replicate effects proportion) across three trial sites (CALL, CARS, and REDE) for the 30 traits assessed in the white spruce population. Abbreviations used for the traits and sites are described in the text.

	Number of trees			Replicate effects proportion		
**Trait**	**CALL**	**CARS**	**REDE**	**CALL**	**CARS**	**REDE**
**HT**	612	311	593	1.9%	8.0%	3.1%
**DBH**	612	311	593	1.7%	8.3%	0.9%
**WD**	591	299	558	5.6%	0.0%	0.5%
**MFA**	610	311	589	0.0%	4.0%	2.9%
**Resistance**	586	296	553	0.5%	1.0%	4.0%
**Sensitivity**	592	298	555	2.6%	4.2%	1.8%
**$\delta^{13}C$**	609	311	589	1.1%	5.5%	0.0%
**α-pinene**	603	244	571	0.0%	0.0%	3.1%
**β-pinene**	492	15	425	1.0%	*b*	2.2%
**camphene**	623	314	603	0.5%	3.9%	2.2%
**camphor**	578	30	575	3.6%	*b*	2.0%
**myrcene**	603	298	571	0.6%	0.0%	2.5%
**limonene**	605	293	574	0.3%	1.4%	1.7%
**terpinolene**	427	3	476	1.4%	*b*	1.7%
**total monoterpenes**	607	308	580	0.9%	0.8%	2.5%
**gallic acid**	418	0	529	19.4%	*b*	49.4%
**gallocatechi**	419	0	529	11.3%	*b*	0.1%
**catechin**	419	0	529	3.9%	*b*	4.3%
**pungenol**	419	0	529	2.3%	*b*	35.0%
**caffeic acid**	419	0	529	4.0%	*b*	12.2%
**vanillin**	419	0	529	20.4%	*b*	36.3%
**taxifolin**	399	0	529	1.0%	*b*	2.4%
**quercetin**	387	0	529	19.7%	*b*	15.0%
**naringenin**	419	0	529	0.7%	*b*	0.5%
**kaempferol**	419	0	529	28.4%	*b*	4.1%
**apigenin**	418	0	529	8.8%	*b*	60.1%
**isorhamnetin**	419	0	529	23.2%	*b*	8.5%
**gs**	324	247	0	7.7%	12.0%	*b*
**WUE**	324	247	0	4.3%	9.9%	*b*
**Ci**	324	247	0	2.7%	10.6%	*b*

NOTE: [b] The proportion of variance of replication effects with respect to the total variance were not calculated due to the unavailability or insufficiency of phenotypic data.

Table S3. Estimated additive genetic and residual variances (and approximate standard errors) for each of the 30 traits assessed in the white spruce population at three progeny test sites. The table also includes the total phenotypic variance (averaged across sites), G × E variance (averaged across sites), and the G × E variance expressed as a percentage of the total phenotypic variance (% G × E variance). Abbreviations used for the traits and sites are described in the text.

	Additive genetic variance			Residual variance			Averaged total variance	Averaged G × E variance[c]	% G × E variance
**Trait / site**	**CALL**	**CARS**	**REDE**	**CALL**	**CARS**	**REDE**			
**HT**	0.78 (0.19)	0.72 (0.32)	0.76 (0.17)	0.01 (0.16)	0.12 (0.28)	0.01 (0.14)	0.80	0.34	42%
**DBH**	0.60 (0.20)	0.05 (0.29)	0.71 (0.18)	0.22 (0.17)	0.90 (0.29)	0.10 (0.15)	0.86	0.19	22%
**WD**	0.48 (0.19)	0.59 (0.31)	0.66 (0.20)	0.40 (0.16)	0.26 (0.27)	0.14 (0.17)	0.85	0.01	1%
**MFA**[a]	0.40 (0.17)	0.27 (0.29)	0.18 (0.15)	0.51 (0.15)	0.66 (0.28)	0.77 (0.15)	0.93	0.08	9%
**Resistance**	0.29 (0.17)	0.16 (0.30)	0.55 (0.20)	0.64 (0.16)	0.81 (0.29)	0.30 (0.18)	0.92	0.17	19%
**Sensitivity**	0.47 (0.18)	0.07 (0.30)	0.69 (0.22)	0.40 (0.16)	0.90 (0.30)	0.16 (0.18)	0.90	0.13	14%
**$\delta^{13}C$**	0.56 (0.16)	0.75 (0.26)	0.73 (0.17)	0.29 (0.14)	0.06 (0.23)	0.03 (0.14)	0.81	0.03	3%
**α-pinene**[a]	0.55 (0.20)	0.10 (0.42)	0.47 (0.19)	0.34 (0.17)	0.87 (0.41)	0.43 (0.17)	0.92	0.15	16%
**β-pinene**[a]	0.32 (0.20)	*b*	0.29 (0.23)	0.58 (0.18)	*b*	0.65 (0.22)	0.93	0.12	13%
**camphene**[a]	0.77 (0.21)	0.14 (0.51)	0.57 (0.18)	0.03 (0.17)	0.84 (0.52)	0.30 (0.16)	0.88	0.20	23%
**camphor**[a]	0.34 (0.19)	*b*	0.53 (0.20)	0.57 (0.17)	*b*	0.34 (0.18)	0.89	0.25	28%
**myrcene**[a]	0.77 (0.19)	0.58 (0.29)	0.47 (0.17)	0.04 (0.16)	0.27 (0.27)	0.42 (0.15)	0.85	0.07	8%
**limonene**[a]	0.65 (0.20)	0.46 (0.32)	0.34 (0.16)	0.21 (0.16)	0.43 (0.29)	0.55 (0.15)	0.88	0.01	2%
**terpinolene**[a]	0.56 (0.21)	*b*	0.31 (0.18)	0.31 (0.19)	*b*	0.63 (0.18)	0.91	0.33	37%

**total monoterpenes**[a]	0.57 (0.19)	0.31 (0.31)	0.59 (0.19)	0.29 (0.16)	0.60 (0.29)	0.28 (0.17)	0.88	0.04	4%
**gallic acid**	0.45 (0.22)	*b*	0.40 (0.18)	0.45 (0.20)	*b*	0.48 (0.17)	0.89	0.21	23%
**gallocatechin**[a]	0.33 (0.23)	*b*	0.60 (0.19)	0.59 (0.21)	*b*	0.20 (0.17)	0.86	0.18	21%
**catechin**	0.24 (0.21)	*b*	0.61 (0.20)	0.70 (0.20)	*b*	0.17 (0.17)	0.86	0.08	9%
**pungenol**[a]	0.18 (0.20)	*b*	0.44 (0.20)	0.77 (0.20)	*b*	0.42 (0.18)	0.90	0.05	6%
**caffeic acid**[a]	0.67 (0.21)	*b*	0.42 (0.19)	0.08 (0.18)	*b*	0.41 (0.17)	0.79	0.11	14%
**vanillin**	0.49 (0.23)	*b*	0.56 (0.20)	0.36 (0.20)	*b*	0.22 (0.18)	0.82	0.29	36%
**taxifolin**[a]	0.27 (0.23)	*b*	0.43 (0.20)	0.66 (0.21)	*b*	0.44 (0.18)	0.90	0.12	14%
**quercetin**[a]	0.21 (0.23)	*b*	0.57 (0.22)	0.75 (0.22)	*b*	0.23 (0.19)	0.88	0.26	29%
**naringenin**[a]	0.59 (0.28)	*b*	0.43 (0.18)	0.27 (0.23)	*b*	0.44 (0.17)	0.86	0.25	29%
**kaempferol**[a]	0.29 (0.23)	*b*	0.18 (0.15)	0.64 (0.21)	*b*	0.73 (0.15)	0.92	0.08	8%
**apigenin**[a]	0.63 (0.25)	*b*	0.36 (0.19)	0.20 (0.21)	*b*	0.54 (0.18)	0.87	0.25	29%
**isorhamnetin**[a]	0.35 (0.24)	*b*	0.52 (0.21)	0.58 (0.21)	*b*	0.28 (0.19)	0.87	0.24	27%
**gs**	0.24 (0.30)	0.14 (0.34)	*b*	0.73 (0.29)	0.80 (0.33)	*b*	0.96	0.07	8%
**WUE**	0.29 (0.26)	0.25 (0.32)	*b*	0.65 (0.25)	0.69 (0.33)	*b*	0.94	0.20	22%
**Ci**	0.28 (0.26)	0.20 (0.32)	*b*	0.66 (0.25)	0.75 (0.33)	*b*	0.95	0.19	20%

NOTE: [a] Logarithmic transformed

[b] Additive and residual variance estimates and their approximate standard errors were not calculated due to the unavailability or insufficiency of phenotypic data

[c] The G × E variance averaged over the three sites was calculated using equation 27 from Itoh and Yamada (1990)

Table S4. Average predictive ability (and approximate standard errors) and prediction bias for each of the 30 traits studied across the three sites and average across sites (Average) and traits (General average). Averages for each site followed by the same letter are not significantly different (α = 0.05) according to the Tukey test. Abbreviations used for the traits and sites are described in the text.

Trait	Predictive ability				Prediction bias			
	CALL	CARS	REDE	Average	CALL	CARS	REDE	Average
**HT**	0.35 (0.03)^{a}	0.19 (0.05)^{b}	0.34 (0.04)^{a}	0.30 (0.05)	1.06 (0.11)^{a}	0.91 (0.24)^{a}	0.98 (0.14)^{a}	0.99 (0.17)
**DBH**	0.28 (0.04)^{a}	0.03 (0.002)^{b}	0.33 (0.03)^{a}	0.21 (0.05)	1.00 (0.14)^{a}	1.01 (0.07)^{a}	1.05 (0.09)^{a}	1.02 (0.10)
**WD**	0.27 (0.02)^{a}	0.26 (0.03)^{a}	0.32 (0.03)^{a}	0.28 (0.03)	1.08 (0.09)^{a}	0.90 (0.11)^{a}	1.01 (0.09)^{a}	1.00 (0.10)
**MFA**	0.18 (0.01)^{a}	0.09 (0.02)^{b}	0.10 (0.01)^{b}	0.12 (0.02)	1.05 (0.07)^{a}	0.89 (0.23)^{a}	0.99 (0.08)^{a}	0.98 (0.14)
**Resistance**	0.13 (0.01)^{a}	0.09 (0.01)^{a}	0.21 (0.02)^{b}	0.14 (0.02)	1.06 (0.08)^{a}	1.03 (0.17)^{a}	0.99 (0.11)^{a}	1.03 (0.12)
**Sensitivity**	0.23 (0.02)^{a}	0.05 (0.002)^{b}	0.28 (0.04)^{a}	0.19 (0.04)	1.06 (0.13)^{a}	1.03 (0.06)^{a}	0.97 (0.16)^{a}	1.02 (0.12)
**$\delta^{13}C$**	0.30 (0.03)^{a}	0.38 (0.05)^{a}	0.41 (0.03)^{a}	0.36 (0.04)	1.00 (0.11)^{a}	1.11 (0.17)^{a}	1.05 (0.09)^{a}	1.06 (0.12)
**α-pinene**	0.23 (0.02)^{a}	0.07 (0.01)^{b}	0.19 (0.02)^{a}	0.16 (0.03)	1.06 (0.11)^{a}	1.08 (0.07)^{a}	1.04 (0.10)^{a}	1.06 (0.09)
**β-pinene**	0.13 (0.02)^{a}	*	0.12 (0.01)^{a}	0.12 (0.02)	1.05 (0.18)^{a}	*	1.12 (0.13)^{a}	1.09 (0.15)
**camphene**	0.32 (0.03)^{a}	0.09 (0.01)^{b}	0.23 (0.02)^{c}	0.21 (0.04)	0.98 (0.10)^{a}	1.00 (0.10)^{a}	0.96 (0.08)^{a}	0.98 (0.09)
**camphor**	0.15 (0.02)^{a}	*	0.21 (0.02)^{a}	0.18 (0.02)	1.09 (0.14)^{a}	*	1.04 (0.08)^{a}	1.06 (0.11)
**myrcene**	0.34 (0.04)^{a}	0.24 (0.03)^{ab}	0.23 (0.02)^{b}	0.27 (0.03)	1.00 (0.11)^{a}	1.01 (0.15)^{a}	0.96 (0.08)^{a}	0.99 (0.11)
**limonene**	0.27 (0.03)^{a}	0.19 (0.04)^{a}	0.20 (0.01)^{a}	0.22 (0.03)	0.95 (0.13)^{a}	0.90 (0.19)^{a}	1.09 (0.09)^{a}	0.98 (0.14)
**terpinolene**	0.20 (0.03)^{a}	*	0.12 (0.02)^{b}	0.16 (0.03)	1.05 (0.16)^{a}	*	1.03 (0.16)^{a}	1.04 (0.15)
**total monoterpenes**	0.27 (0.03)^{a}	0.16 (0.01)^{a}	0.25 (0.05)^{a}	0.23 (0.04)	1.08 (0.17)^{a}	1.06 (0.12)^{a}	1.05 (0.24)^{a}	1.06 (0.18)
**gallic acid**	0.16 (0.02)^{a}		0.16 (0.02)^{a}	0.16 (0.02)	1.00 (0.13)^{a}	*	1.00 (0.12)^{a}	1.00 (0.12)
**gallocatechin**	0.14 (0.02)^{a}	*	0.27 (0.02)^{b}	0.20 (0.03)	1.00 (0.12)^{a}	*	1.23 (0.21)^{a}	1.11 (0.17)
**catechin**	0.14 (0.01)^{a}	*	0.29 (0.03)^{b}	0.21 (0.03)	1.13 (0.07)^{a}	*	1.18 (0.18)^{a}	1.16 (0.13)
**pungenol**	0.10 (0.01)^{a}	*	0.17 (0.02)^{b}	0.13 (0.02)	1.03 (0.11)^{a}	*	0.99 (0.13)^{a}	1.01 (0.12)
**caffeic acid**	0.37 (0.03)^{a}	*	0.22 (0.02)^{b}	0.30 (0.04)	1.18 (0.14)^{a}	*	1.11 (0.14)^{a}	1.14 (0.13)
**vanillin**	0.19 (0.03)^{a}	*	0.26 (0.02)^{b}	0.22 (0.03)	1.03 (0.14)^{a}	*	1.11 (0.11)^{a}	1.07 (0.12)
**taxifolin**	0.11 (0.01)^{a}	*	0.18 (0.02)^{b}	0.14 (0.02)	1.08 (0.13)^{a}	*	1.08 (0.14)^{a}	1.08 (0.13)
**quercetin**	0.08 (0.01)^{a}	*	0.24 (0.02)^{b}	0.16 (0.03)	1.01 (0.15)^{a}	*	1.07 (0.11)^{a}	1.04 (0.13)
**naringenin**	0.22 (0.04)^{a}	*	0.18 (0.02)^{a}	0.20 (0.03)	1.03 (0.20)^{a}	*	1.06 (0.17)^{a}	1.05 (0.18)
**kaempferol**	0.13 (0.01)^{a}	*	0.12 (0.01)^{a}	0.13 (0.01)	1.05 (0.13)^{a}	*	1.48 (0.11)^{b}	1.26 (0.13)
**apigenin**	0.29 (0.02)^{a}	*	0.15 (0.01)^{b}	0.22 (0.03)	1.22 (0.11)^{a}	*	1.04 (0.08)^{a}	1.13 (0.10)
**isorhamnetin**	0.13 (0.02)^{a}	*	0.23 (0.03)^{b}	0.18 (0.03)	0.99 (0.11)^{a}	*	1.14 (0.19)^{a}	1.06 (0.15)
**gs**	0.05 (0.02)^{a}	0.07 (0.01)^{a}	*	0.06 (0.01)	0.64 (0.25)^{a}	1.32 (0.18)^{b}	*	0.98 (0.24)
**WUE**	0.09 (0.02)^{a}	0.07 (0.02)^{a}	*	0.08 (0.02)	0.88 (0.15)^{a}	1.10 (0.27)^{a}	*	0.99 (0.22)
**Ci**	0.09 (0.01)^{a}	0.05 (0.01)^{b}	*	0.07 (0.01)	0.98 (0.15)^{a}	0.98 (0.28)^{a}	*	0.98 (0.22)
**General average**	**0.20 (0.04)**^{a}	**0.13 (0.04)**^{b}	**0.22 (0.03)**^{c}	**0.19 (0.04)**	**1.03 (0.13)**^{a}	**1.02 (0.17)**^{a}	**1.07 (0.13)**^{a}	**1.04 (0.14)**

NOTE: *Predictive ability and prediction bias (and their approximate standard errors) were not calculated due to the unavailability or insufficiency of phenotypic data.

Fig S1. Density distribution for the studied traits in white spruce in each of the three test sites. Logarithmic transformations were applied to MFA, as well as all monoterpene and polyphenolic compounds, except for gallic acid, catechin, and vanillin to improve data normality. Abbreviations used for the traits and sites are described in the text.

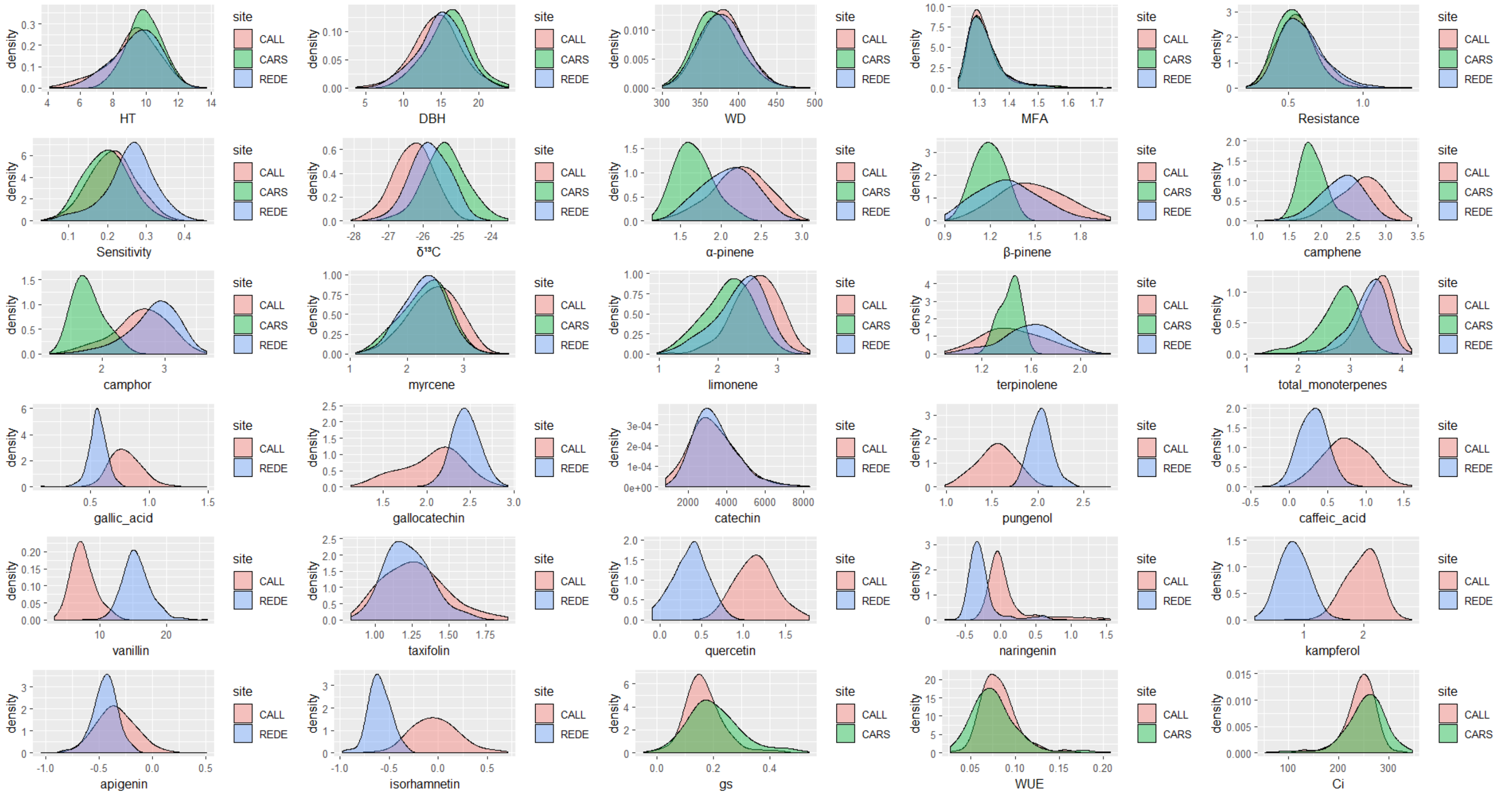

Fig S2. Heat map of the pair-wise relationship coefficients among the 1,540 genotyped white spruce trees. The heat scale represents pair-wise relationship coefficients for all pairs of individual trees. Small squares near the diagonal elements represent trees within the same family across the three sites. The small squares that form lines in the off-diagonal elements indicate trees from the same family but located at different sites.

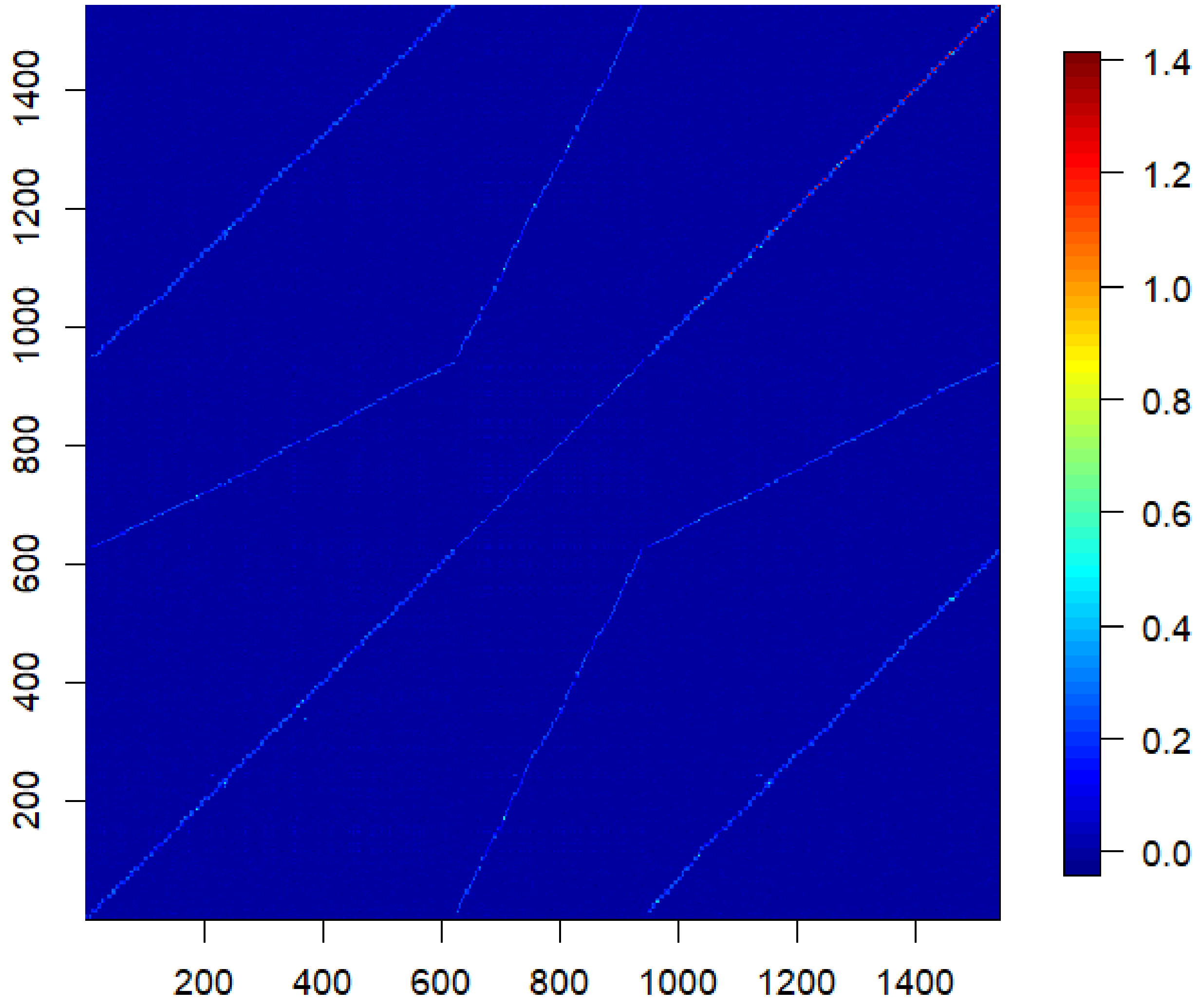

Fig S3. Histogram of the diagonal (a) and off-diagonal (b) elements of the genomic relationship *G*-matrix. For better visualization of the off-diagonal elements of the ***G***-matrix we excluded coefficients smaller than 0.009.

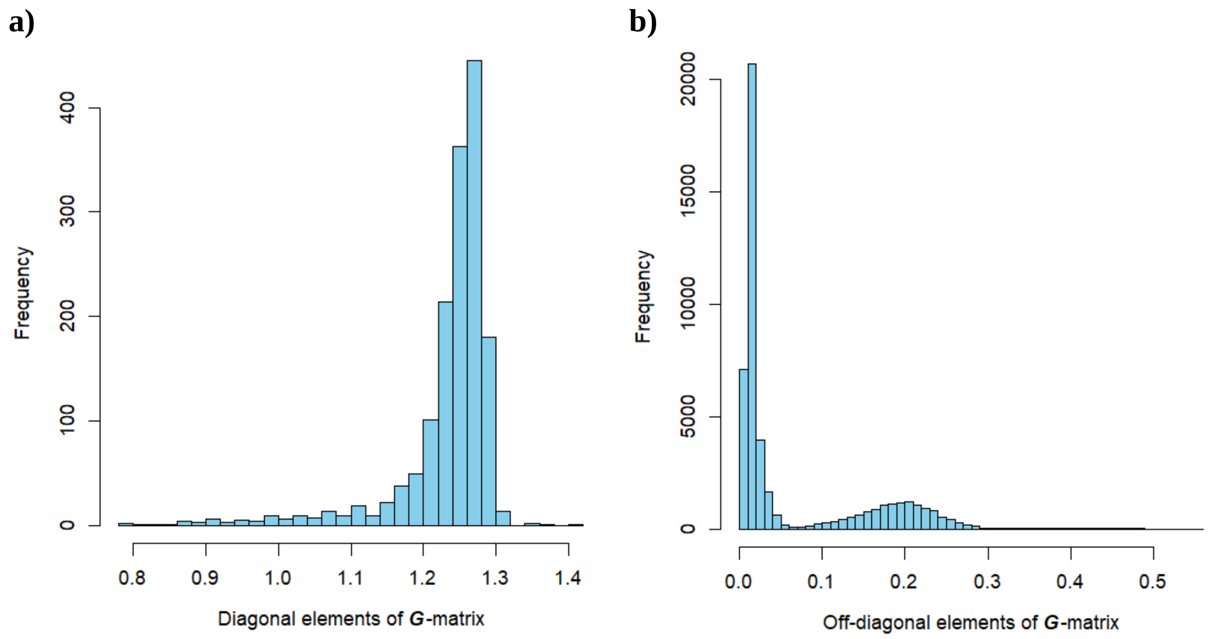

Fig S4. Estimated genetic correlations between the three progeny sites for each of the 30 traits assessed in the white spruce population. Genetic correlation estimates are shown in each cell below the diagonal, with colour and size of circle reflecting the genetic correlation strength. The small (weaker) and larger (stronger) circles indicate the strength of the correlation, shown above diagonal. Abbreviations used for the traits and sites are described in the text.

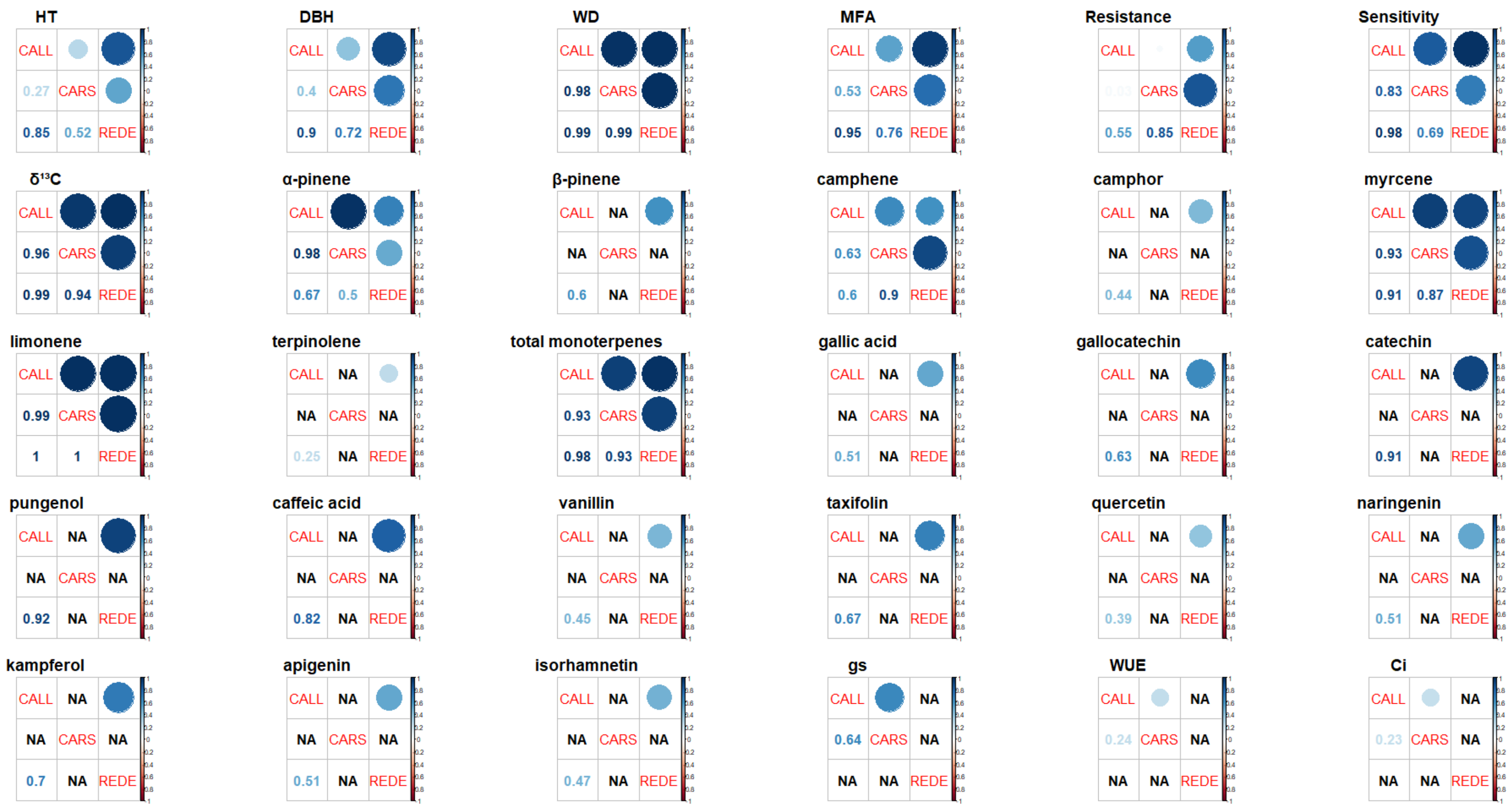

NA = Correlations were not estimated with the CARS and REDE sites due to the unavailability or insufficiency of phenotypic data.

Fig S5. Manhattan plots for the multi-environment genome-wide association (GWAS) analyses for each of the 18 traits studied across two sites (CALL and REDE or CALL and CARS) in the white spruce population. The vertical *y*-axis indicates -log10(*p*-value) and the horizontal *x*-axis indicates the single-SNPs expressed in thousands of SNPs. The red dashed line represents the cutoff *p*-values of 1.07×10^{-07} based on adjusted Bonferroni correction (---; -log10(*p*-value) equal to 6.97) and the blue dashed line represents the cutoff *p*-values of 1.07×10^{-05} (---; -log10(*p*-value) equal to 4.97). Abbreviations used for the traits and sites are described in the text.

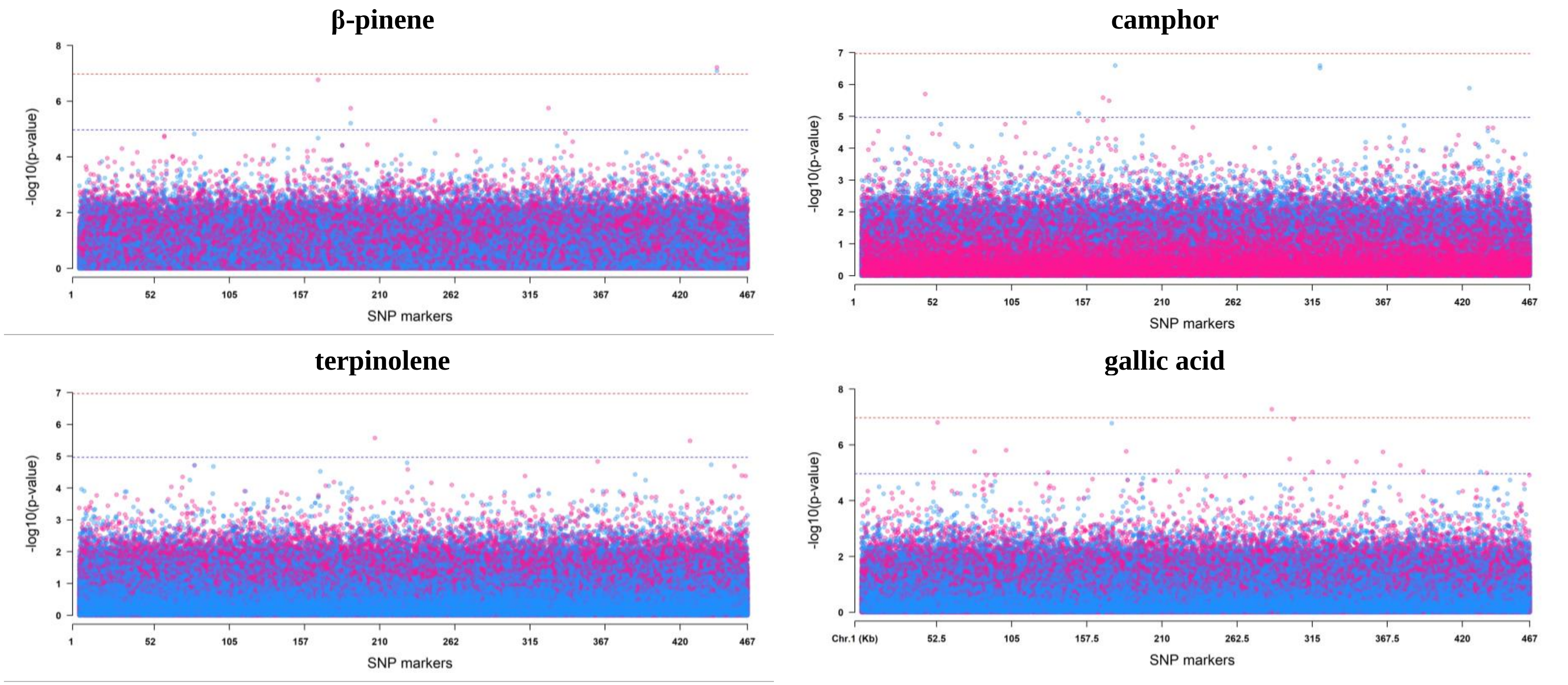

gallocatchin

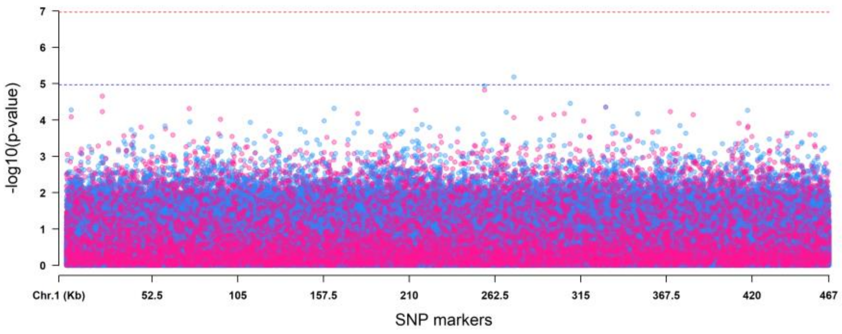

catechin

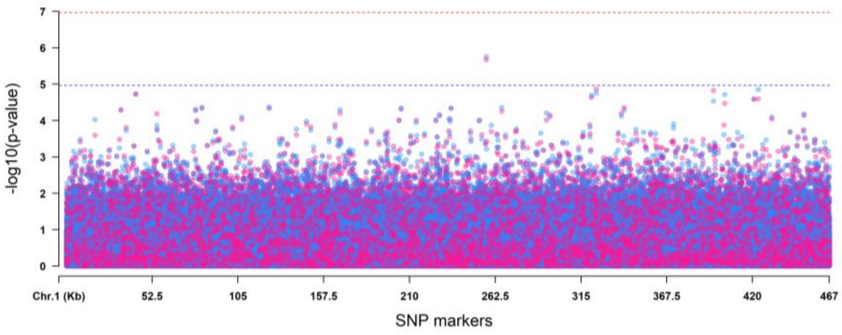

pungenol

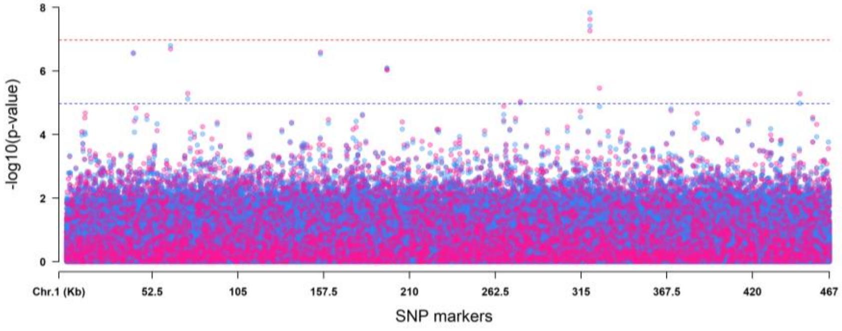

caffeic acid

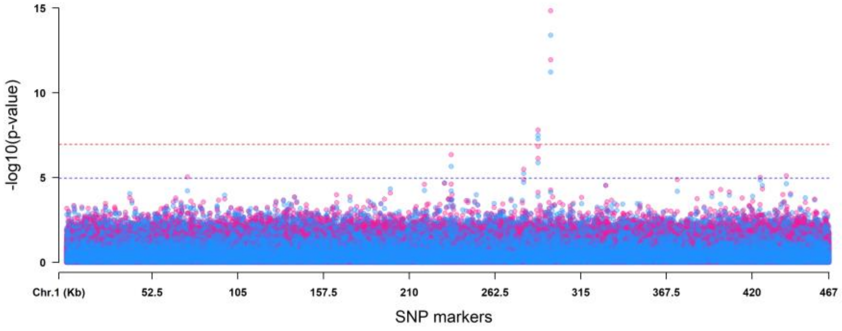

vanillin

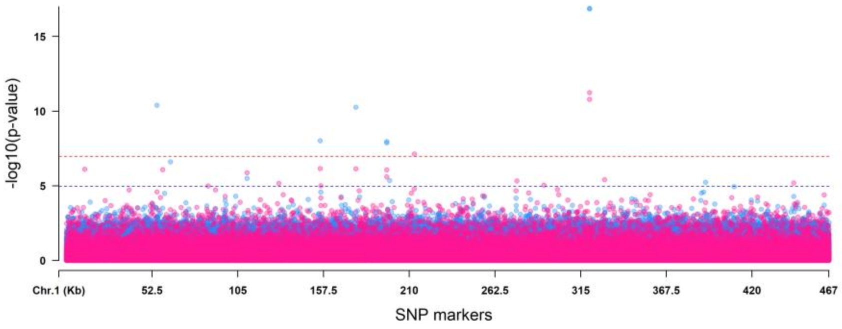

taxifolin

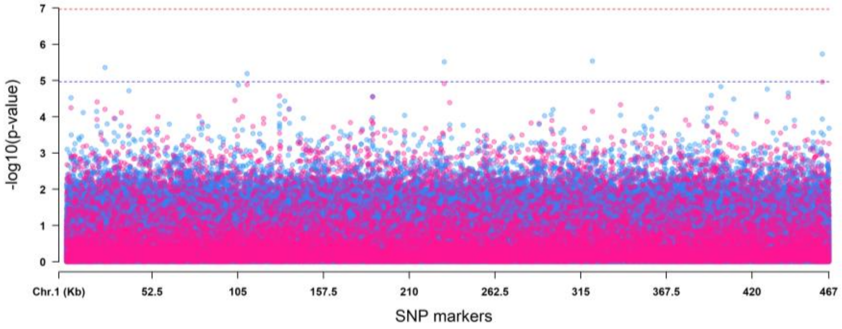

quercetin

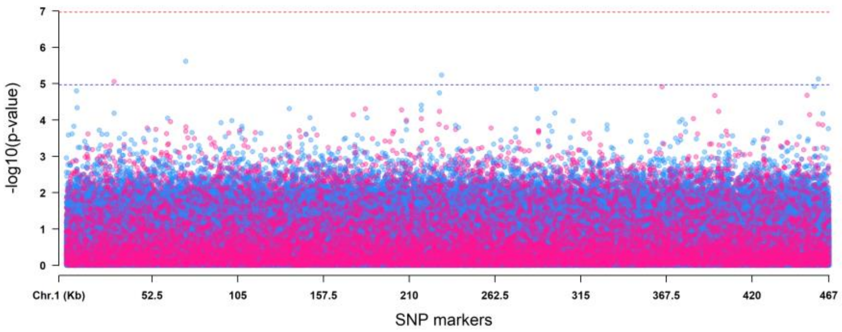

naringenin

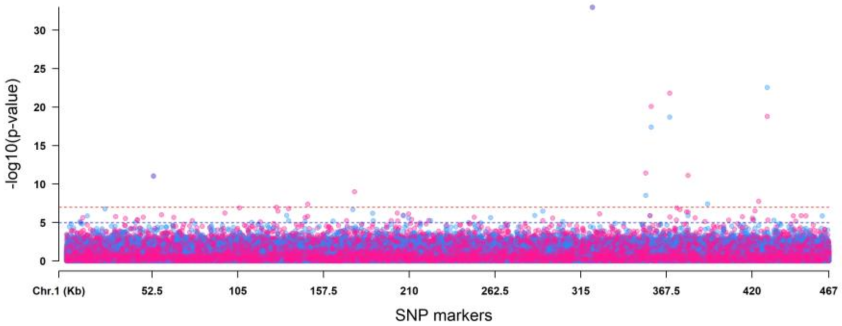

kaempferol

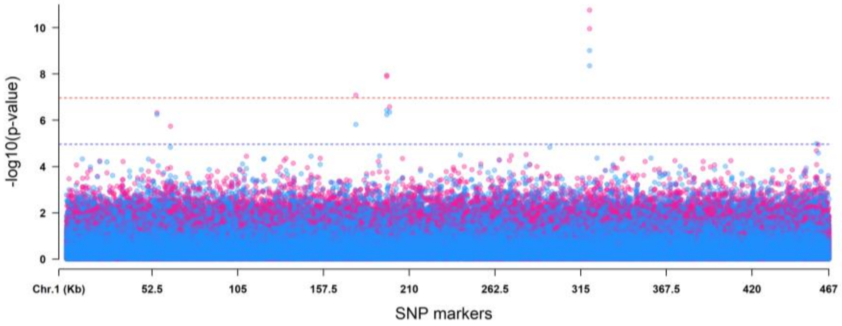

apigenin

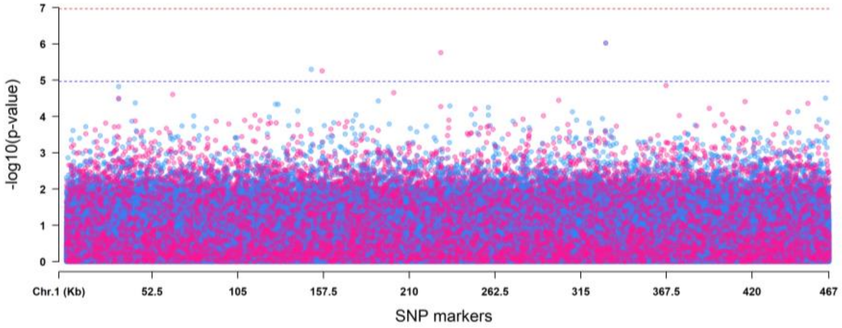

isorhamnetin

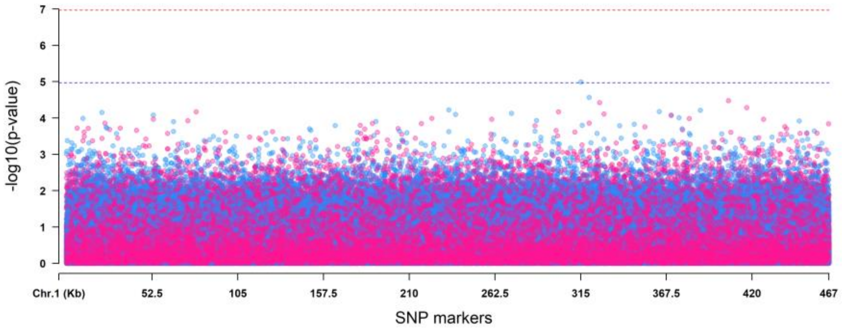

gs

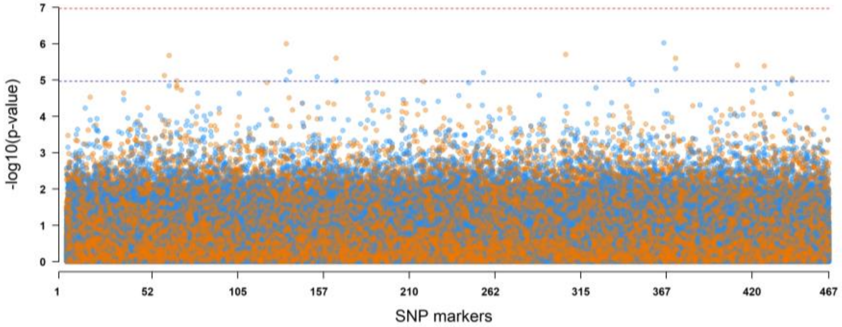

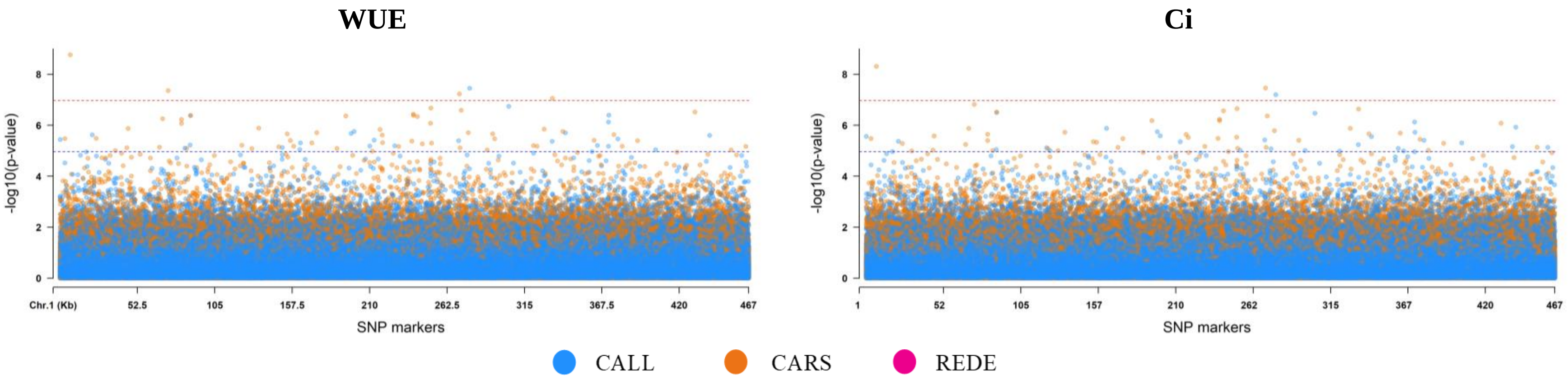
WUE
Ci
-log10(p-value)
0
2
4
6
8
Chr.1 (Kb)
52.5
105
157.5
210
262.5
315
367.5
420
467
1
52
105
157
210
262
315
367
420
467
SNP markers
SNP markers
CALL
CARS
REDE

Fig S6. Venn diagram illustrating the overlap of significant genetic associations (suggestive cutoff *p*-values of 1.07 × 10^{-05}) across two different sites (CALL and REDE or CALL and CARS). The numbers within the diagram indicate the count of SNPs significantly associated with the trait in each environment. Overlapping regions show the number of SNPs that are commonly associated across two environments, highlighting the consistency or variability of genetic effects. Abbreviations used for the traits and sites are described in the text.

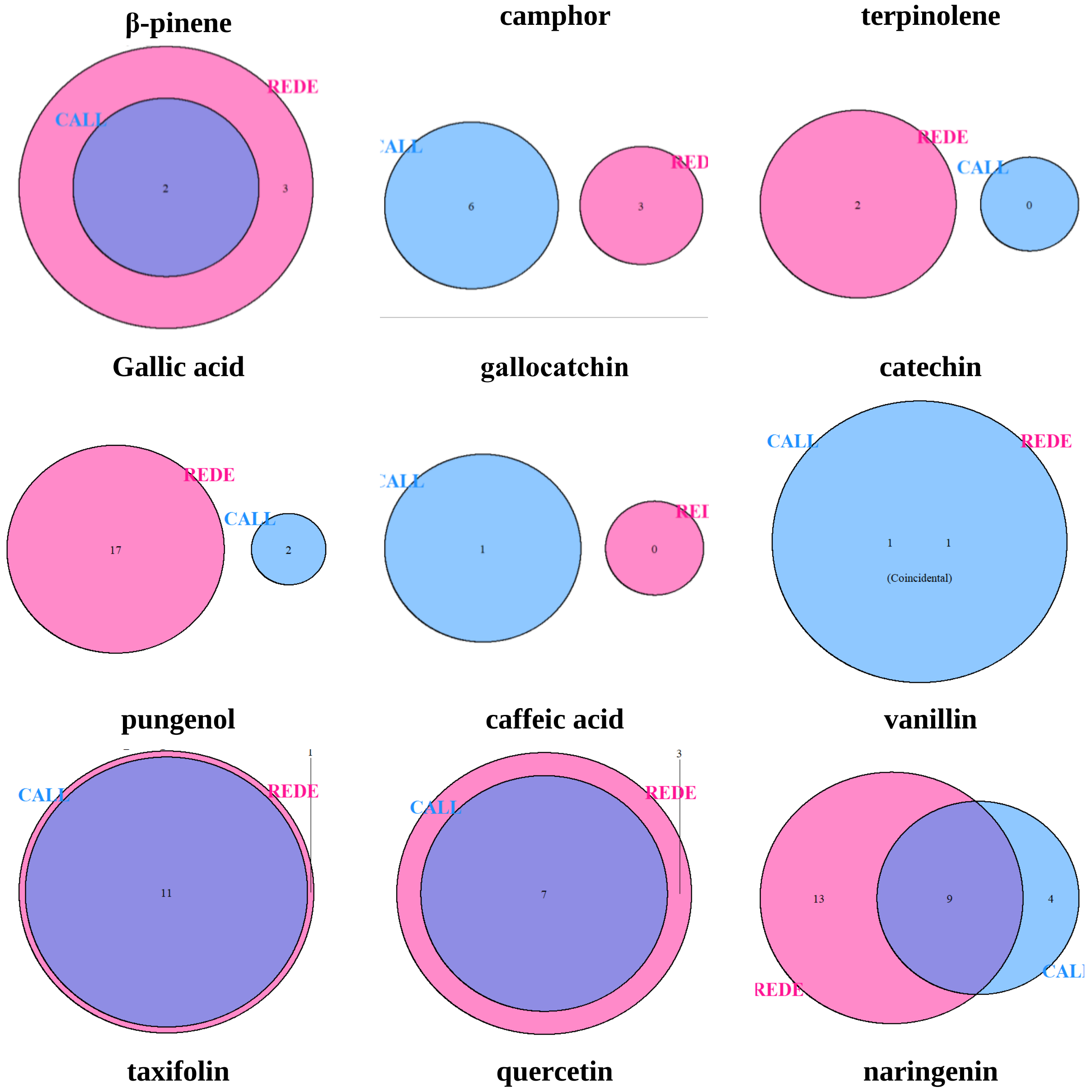

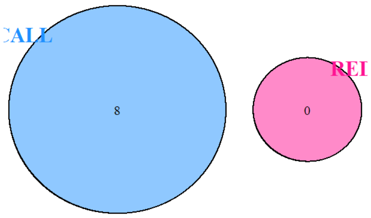

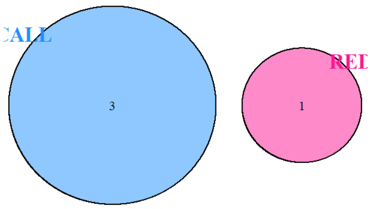

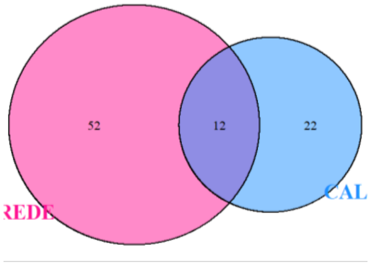

kaempferol **apigenin** **isorhamnetin**

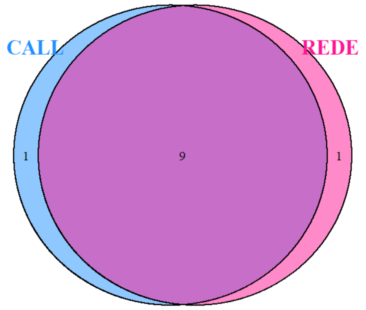

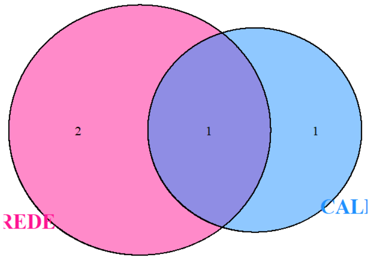

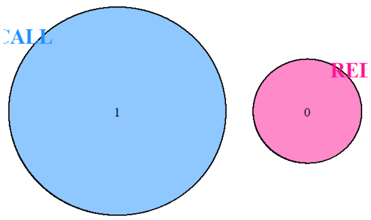

gs **WUE** **Ci**

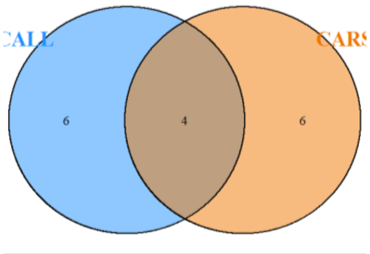

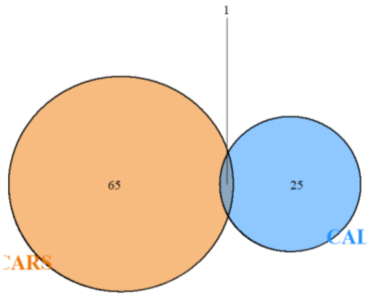

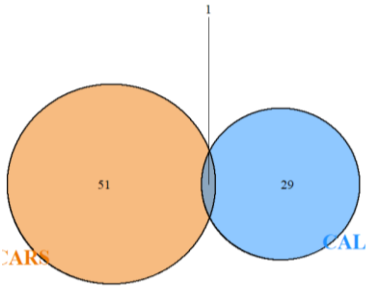

Fig S7. Quantile-quantile (Q-Q) plots for the multi-environment genome-wide association (GWAS) analyses for each of the 30 traits studied across the three sites (CALL, CARS, and REDE) in the white spruce population. Q-Q plot is used to assess the number and magnitude of observed associations between genotyped SNPs and traits under study, compared to the association statistics expected under the null hypothesis of no association. Abbreviations used for the traits and sites are described in the text.

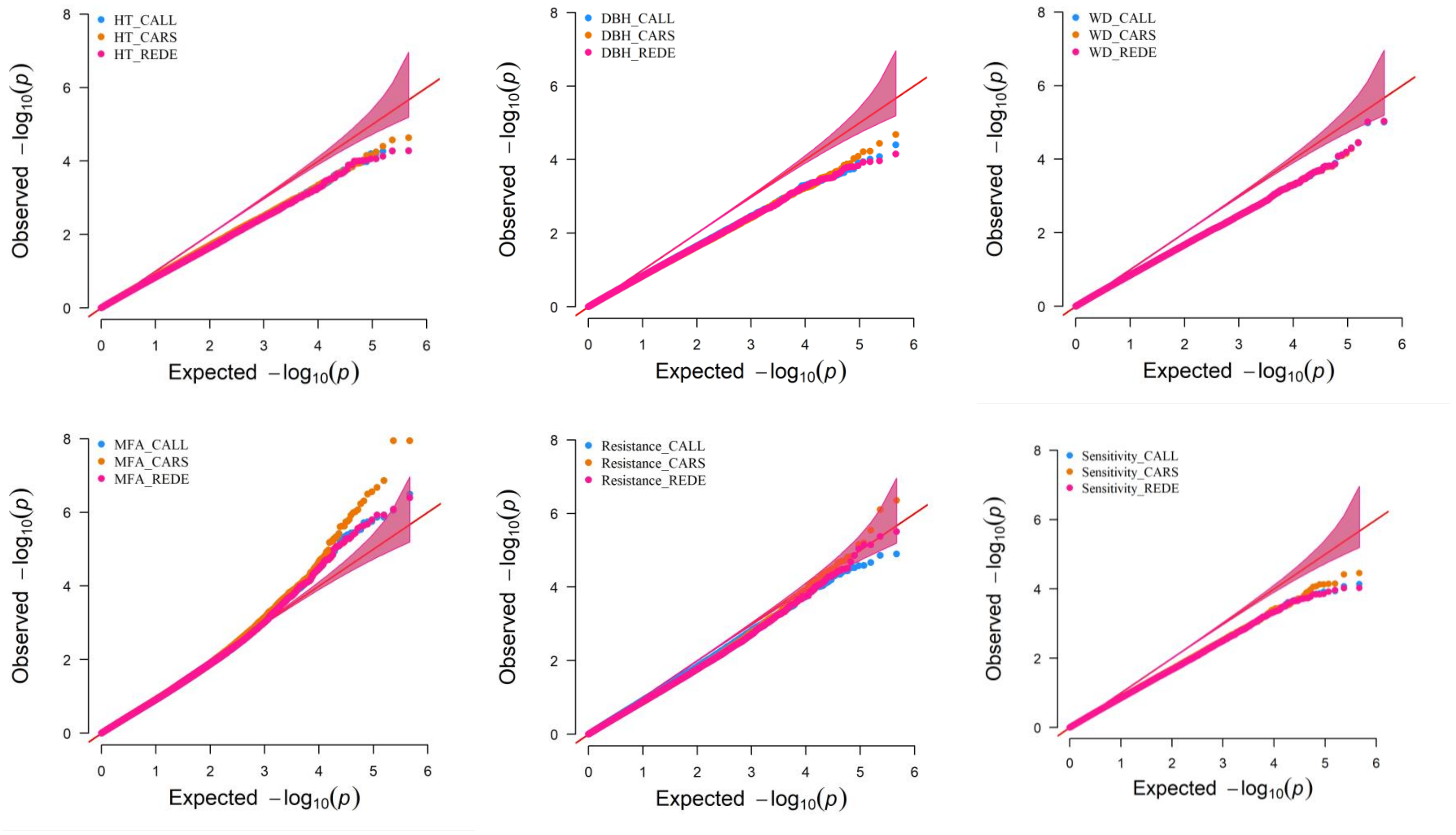

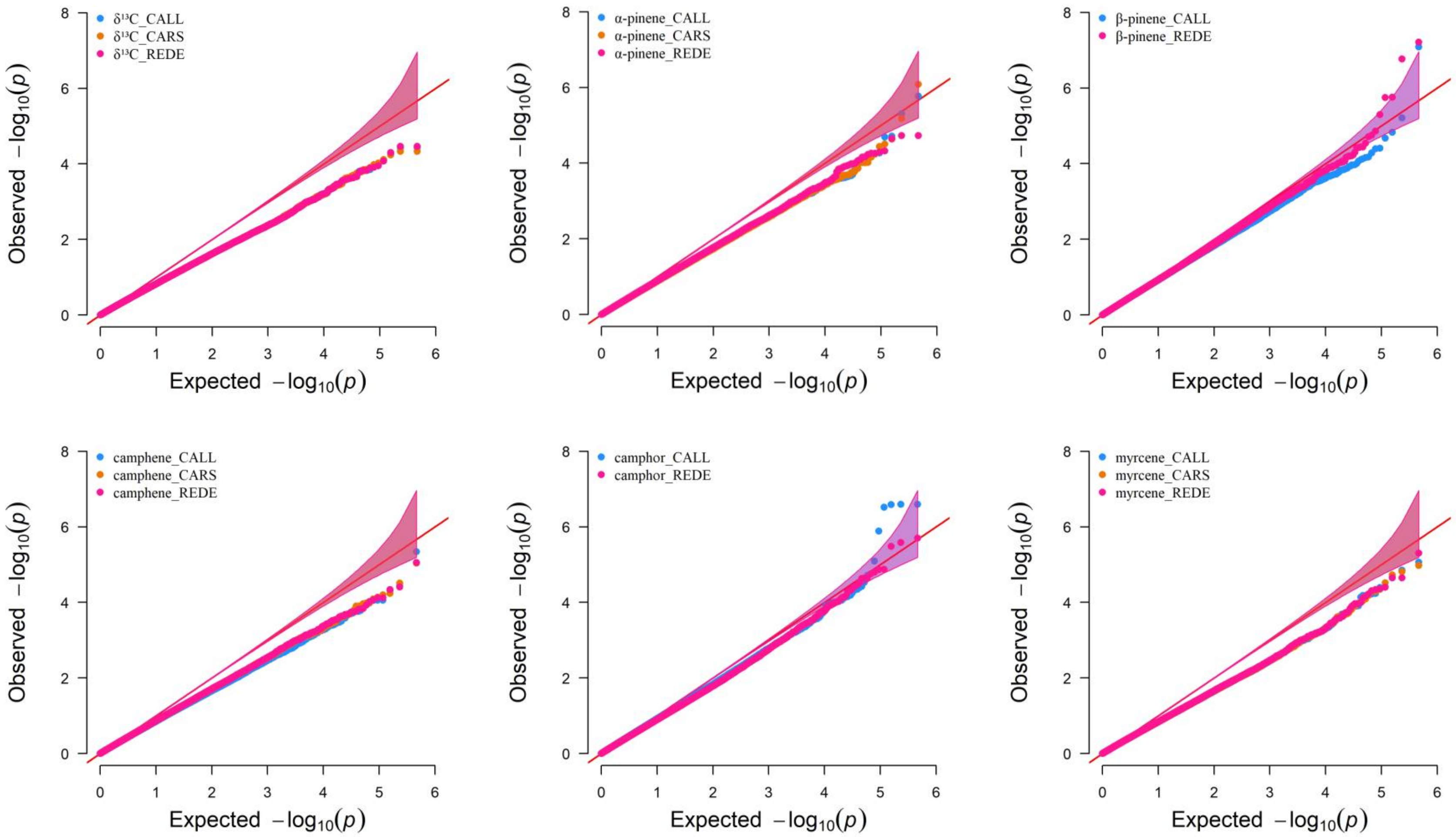
δ13C_CALL
δ13C_CARS
δ13C_REDE
α-pinene_CALL
α-pinene_CARS
α-pinene_REDE
β-pinene_CALL
β-pinene_REDE
camphene_CALL
camphene_CARS
camphene_REDE
camphor_CALL
camphor_REDE
myrcene_CALL
myrcene_CARS
myrcene_REDE
Observed −log10(p)
Expected −log10(p)

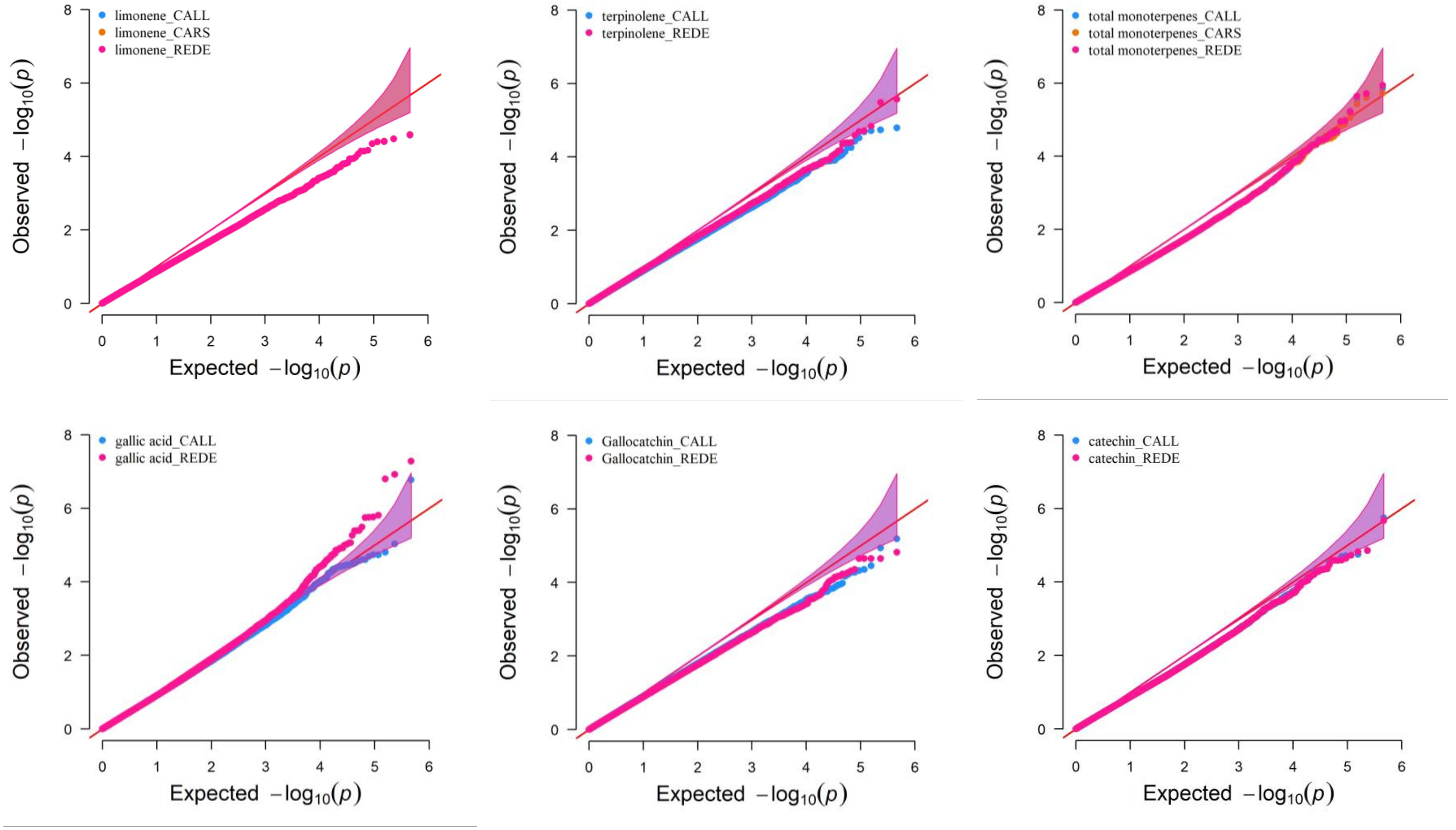
limonene_CALL
limonene_CARS
limonene_REDE
terpinolene_CALL
terpinolene_REDE
total monoterpenes_CALL
total monoterpenes_CARS
total monoterpenes_REDE
gallic acid_CALL
gallic acid_REDE
Gallocatchin_CALL
Gallocatchin_REDE
catechin_CALL
catechin_REDE
Observed $-\log_{10}(p)$
Expected $-\log_{10}(p)$

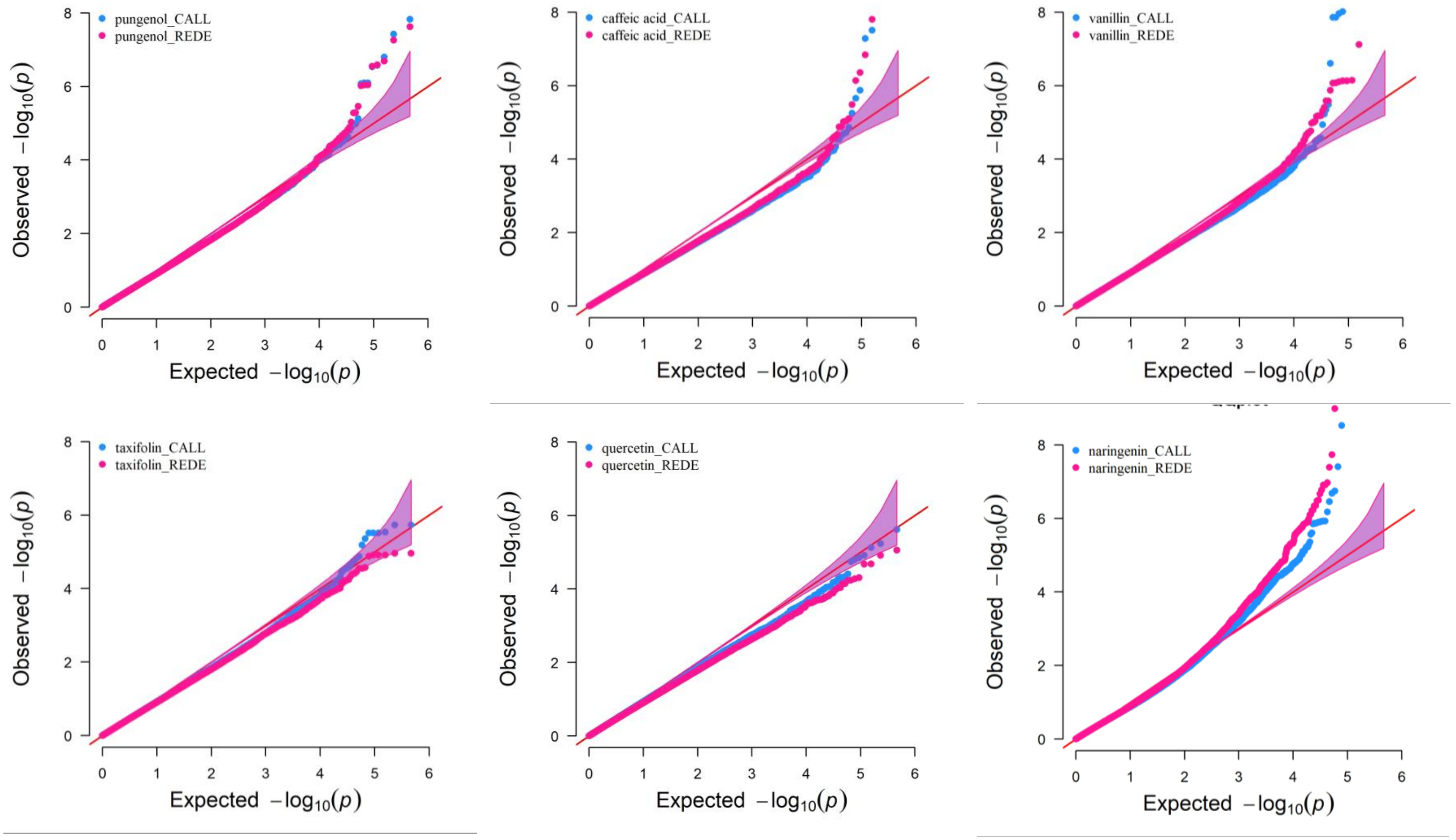
pungenol_CALL
pungenol_REDE
caffeic acid_CALL
caffeic acid_REDE
vanillin_CALL
vanillin_REDE
taxifolin_CALL
taxifolin_REDE
quercetin_CALL
quercetin_REDE
naringenin_CALL
naringenin_REDE
Observed $-\log_{10}(p)$
Expected $-\log_{10}(p)$

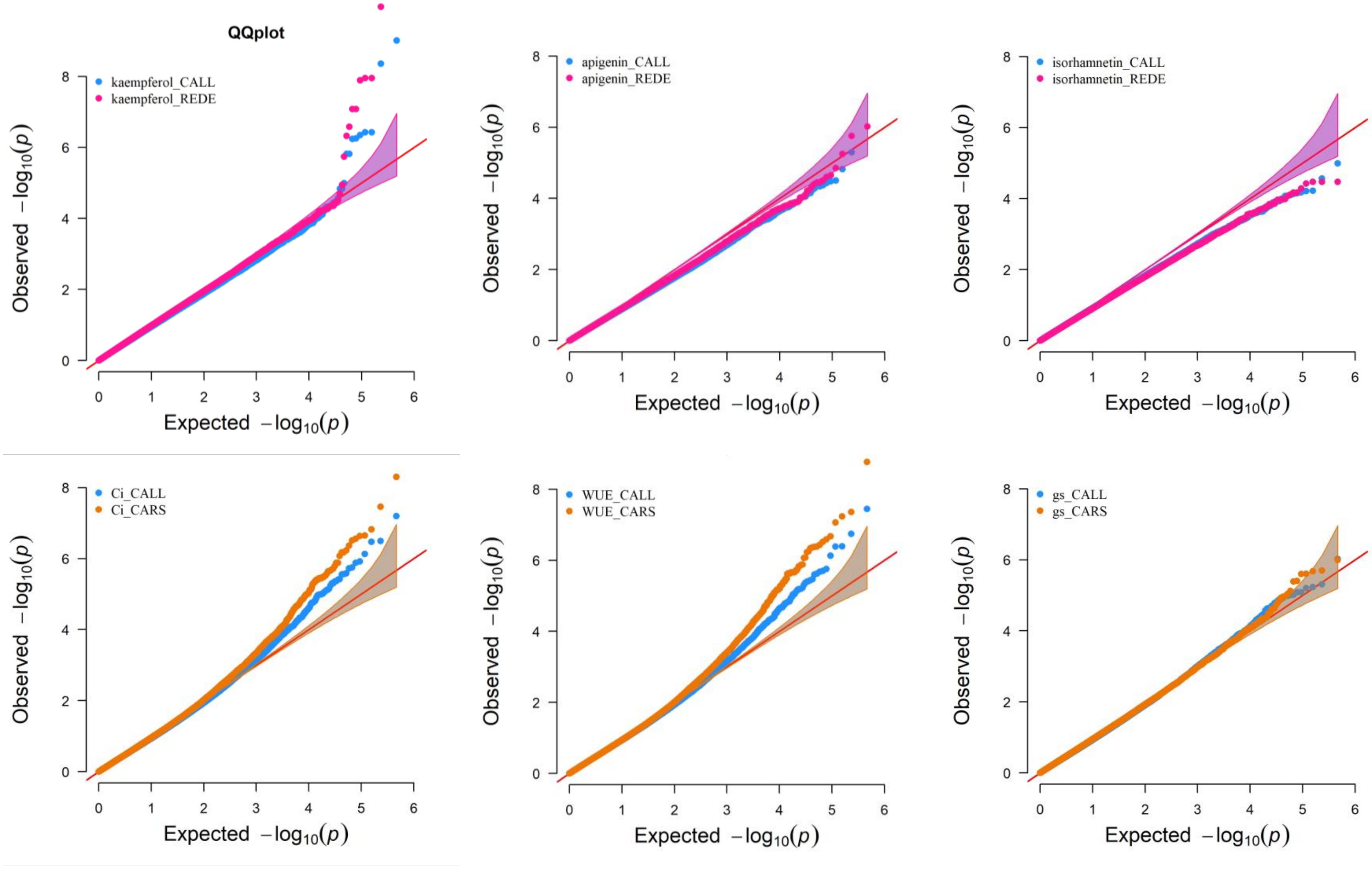
QQplot
kaempferol_CALL
kaempferol_REDE
apigenin_CALL
apigenin_REDE
isorhamnetin_CALL
isorhamnetin_REDE
Ci_CALL
Ci_CARS
WUE_CALL
WUE_CARS
gs_CALL
gs_CARS
Observed −log10(p)
Expected −log10(p)

Fig S8. Relationship between genetic correlation between sites and Spearman rank correlation between the *p*-values of the different pair of sites for the 30 studied traits in the white spruce population. Abbreviations used for the sites are described in the text.

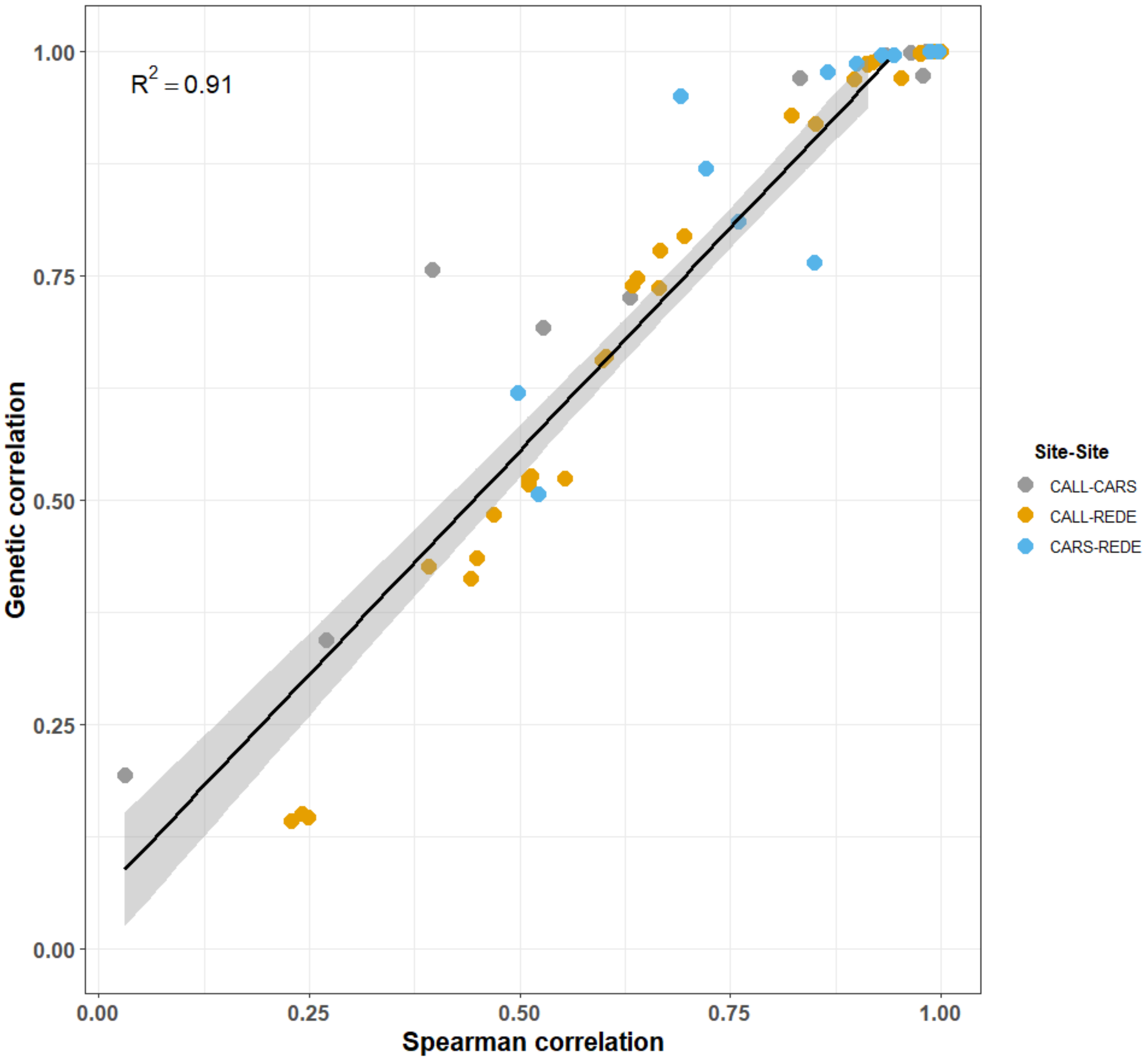

Fig S9. Average predictive ability for each of the 18 traits studied across two sites (CALL and REDE or CALL and CARS). Common letters above box-plots are not significantly different (α = 0.05). Abbreviations used for the traits and sites are described in the text.

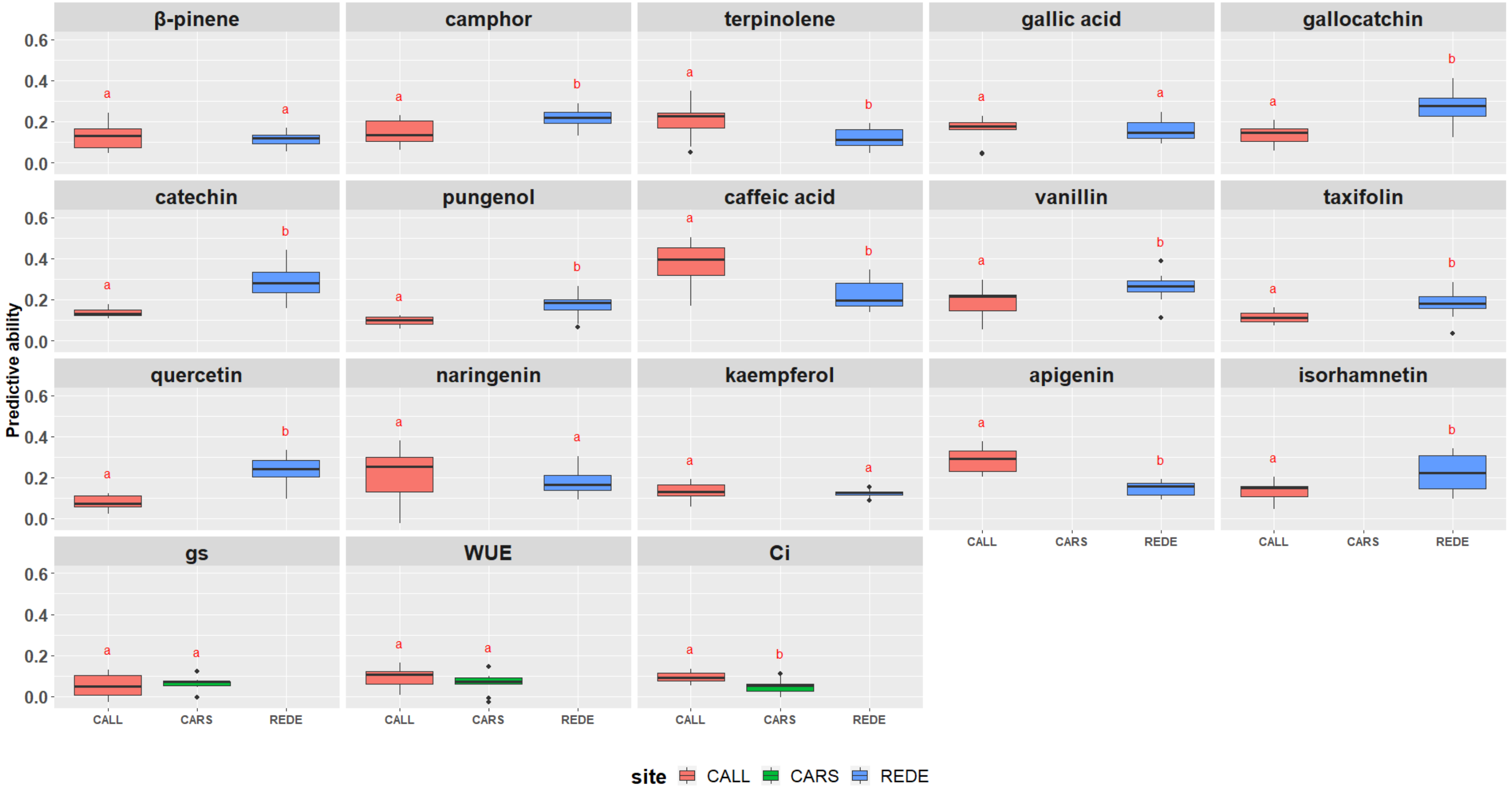

Fig S10. Average prediction bias for each of the 18 traits studied across two sites (CALL and REDE or CALL and CARS). Common letters above box-plots are not significantly different (α = 0.05). Abbreviations used for the traits and sites are described in the text.

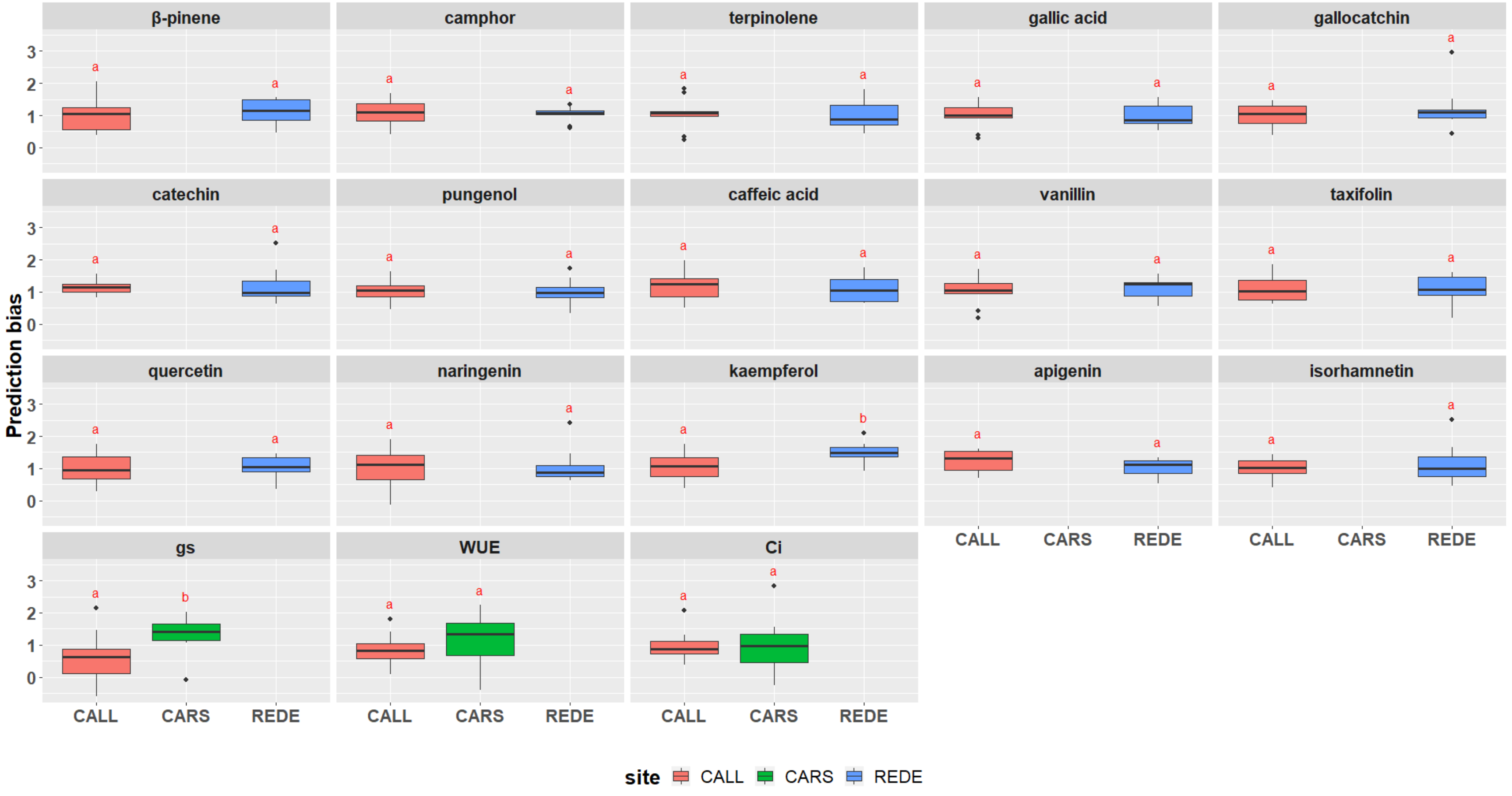

77 **Fig S11. Correlation between predictive ability and narrow-sense heritability for the 30 traits**
78 **in the white spruce population.**

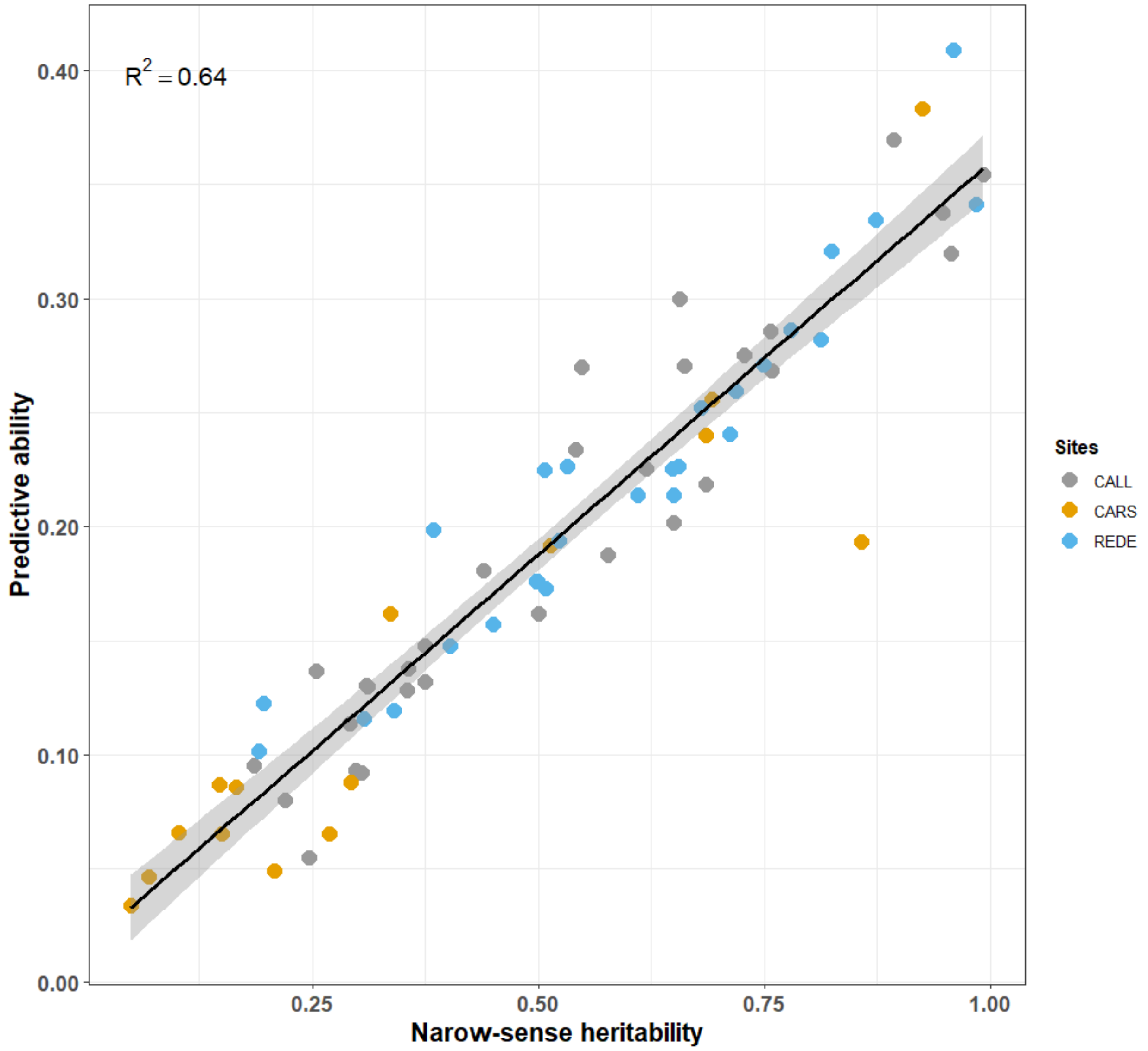

79
